# Electron Flow From the Inner Membrane Towards the Cell Exterior in *Geobacter sulfurreducens*: Biochemical Characterization of Cytochrome CbcL

**DOI:** 10.3389/fmicb.2022.898015

**Published:** 2022-05-10

**Authors:** Jorge M. A. Antunes, Marta A. Silva, Carlos A. Salgueiro, Leonor Morgado

**Affiliations:** ^1^Associate Laboratory i4HB – Institute for Health and Bioeconomy, NOVA School of Science and Technology, NOVA University Lisbon, Caparica, Portugal; ^2^UCIBIO – Applied Molecular Biosciences Unit, Chemistry Department, NOVA School of Science and Technology, NOVA University Lisbon, Caparica, Portugal

**Keywords:** *Geobacter*, extracellular electron transfer, inner membrane associated cytochrome, protein-protein interactions, NMR

## Abstract

Exoelectrogenic microorganisms are in the spotlight due to their unique respiratory mechanisms and potential applications in distinct biotechnological fields, including bioremediation, bioenergy production and microbial electrosynthesis. These applications rely on the capability of these microorganisms to perform extracellular electron transfer, a mechanism that allows the bacteria to transfer electrons to the cell’s exterior by establishing functional interfaces between different multiheme cytochromes at the inner membrane, periplasmic space, and outer membrane. The multiheme cytochrome CbcL from *Geobacter sulfurreducens* is associated to the inner membrane and plays an essential role in the transfer of electrons to final electron acceptors with a low redox potential, as Fe(III) oxides and electrodes poised at −100 mV. CbcL has a transmembranar di-heme *b*-type cytochrome domain with six helices, linked to a periplasmic cytochrome domain with nine *c*-type heme groups. The complementary usage of ultraviolet-visible, circular dichroism and nuclear magnetic resonance permitted the structural and functional characterization of CbcL’s periplasmic domain. The protein was found to have a high percentage of disordered regions and its nine hemes are low-spin and all coordinated by two histidine residues. The apparent midpoint reduction potential of the CbcL periplasmic domain was determined, suggesting a thermodynamically favorable transfer of electrons to the putative redox partner in the periplasm − the triheme cytochrome PpcA. The establishment of a redox complex between the two proteins was confirmed by probing the electron transfer reaction and the molecular interactions between CbcL and PpcA. The results obtained show for the first time how electrons are injected into the periplasm of *Geobacter sulfurreducens* for subsequent transfer to the cell’s exterior.

## Introduction

Microorganisms are the most important biogeochemical agents affecting the chemistry, distribution, and the bioavailability of almost all elements (Popescu et al., 2002). A wide group called dissimilatory metal reducing bacteria (DMRB) plays an important role, namely in the biogeochemical cycles of transition metals as chromium, iron, uranium and manganese (Cavalier-Smith et al., 2006). DMRB can couple their oxidative metabolism to the reduction of extracellular metals like Fe(III), U(VI) or Mn(IV) oxides, as well as electrode surfaces. Unlike the most common respiratory pathways, in which a soluble terminal electron acceptor is reduced inside the cell, DMRB developed an electron transport chain capable of transfering the electrons from inside the cell to the exterior (Nealson and Saffarini, 1994). The best characterized DMRB belong to the *Geobacteraceae* and *Shewanellaceae* families, particularly the bacterium *Geobacter sulfurreducens* that produces the highest power densities of all known exoelectrogenic microorganisms (Vasyliv et al., 2013; Logan et al., 2019). The abundance of *c*-type cytochromes encoded in *G. sulfurreducens* genome provides a unique respiratory flexibility (Lovley et al., 2011; Speers and Reguera, 2012). This flexibility can be explored for different applications including the reduction of soluble metals (e.g., Cr(VI), U(VI)) to insoluble precipitates (Cr(III) and U(IV)) in contaminated waters, or the decomposition of hydrocarbon contaminants in soils, which simplifies the bioremediation process of these pollutants (Lovley et al., 2004, 2011). The transport of electrons to the cells’ exterior, a mechanism called extracellular electron transfer (EET), led to the development of different biotechnological applications using bioelectrochemical systems (BES) (Kumar et al., 2015). Microbial fuel cells (MFC), which can couple the oxidation of organic compounds to the production of electric current are the best known BESs, establishing a promising synergy between green energy production and wastewater treatment (Koffi and Okabe, 2020). However, BESs are not limited to MFCs. Microbial electrolysis cells that produce biohydrogen, or microbial desalination cells that desalinate sea water, are also blooming (Al-Amshawee et al., 2020). This wide range of applications makes important to understand the functional mechanism of EET. Studies based on gene-knockout and proteomic analysis in different growth conditions constantly provide new findings that bring more insights on the proteins involved in the EET pathways (Levar et al., 2014; Zacharoff et al., 2016). To date it is well known that these respiratory pathways in *G. sulfurreducens* encompass different multiheme cytochromes along the inner membrane, periplasmic space, and outer membrane (Ueki, 2021). One remarkable finding was that EET in *G. sulfurreducens* could only be described when the contribution of multiple governing redox processes was considered (Marsili et al., 2010; Yoho et al., 2014; Rimboud et al., 2015). In 2014, Levar and co-workers showed that a strain without the gene that encodes for the inner membrane cytochrome ImcH (Δ*imcH*) lost the ability to reduce the electron acceptors Fe(III)-EDTA, Fe(III) citrate, and insoluble Mn(IV) oxides – all with reduction potentials above −100 mV (Levar et al., 2014). However, the Δ*imcH* mutant was still able to reduce Fe(III) oxides with reduction potentials below −100 mV. These results, in agreement with the hypothesis that distinct routes are necessary to describe EET, suggested that at least one alternative quinone oxidoreductase should be active to permit the growth of the mutated *G. sulfurreducens* strain in the presence of electron acceptors with low redox potentials. This was further confirmed by Zacharoff and co-workers by the deletion of a gene coding for another inner membrane cytochrome [*c*- and *b*-type cytochrome for low potential (CbcL)] and the concomitant inability of the mutated strain to reduce low potential electron acceptors, such as Fe(III) oxides and electrodes poised at −100 mV (Zacharoff et al., 2016). Finally, and more recently, Joshi and co-workers described a third inner membrane cytochrome, designated CbcBA, that was shown to be necessary in the final stages of Fe(III) reduction (Joshi et al., 2021). A *G. sulfurreducens* strain lacking this multiheme cytochrome ceased Fe(III) reduction at −210 mV and couldn’t perform electron transfer to electrodes between −210 and −280 mV.

The present work focuses on the cytochrome CbcL which is constituted by two domains: a membrane domain with six transmembrane helices and two *b*-type heme groups, and a periplasmic domain containing nine *c*-type heme groups. The periplasmic domain of cytochrome CbcL was successfully produced and its characterization was obtained by the complementary usage of different spectroscopic techniques, including UV-visible, circular dichroism (CD) and nuclear magnetic resonance (NMR). Interaction studies between cytochrome CbcL and the triheme cytochrome PpcA, one of its putative redox partners in the periplasm, were also monitored by NMR.

## Materials and Methods

### Cloning

DNA sequence of the gene *cbcL* (GSU0274, GenBank accession number AAR33608.1) was obtained from *G. sulfurreducens* PCA genome database from Kyoto Encyclopedia of Genes and Genomes database (Kanehisa et al., 2016). Residues 30-279 (CbcL periplasmic domain) were amplified from genomic DNA using Phusion DNA polymerase (Thermo Scientific) together with primers with restriction sites for NotI and HindIII enzymes. The resulting DNA fragment and vector pVA203 (Londer et al., 2002; Pokkuluri et al., 2004a) were digested, the E-gel^®^ electrophoresis system (Invitrogen) was used to purify them, and T4 DNA ligase (Thermo Scientific) was used to ligate the DNA fragment to the vector. The plasmid was further modified by the addition of a C-terminal Strep-tag^®^. Plasmids were propagated in *Escherichia coli* DH5α cells and colony screenings performed by PCR using Taq DNA polymerase (VWR). Positive clones were cultured in liquid medium. The plasmids were then purified using the NZYMiniprep kit (NZYTech) and sequenced by STABVida (Caparica, Portugal). The resulting plasmid pVA203-CbcL-St codes for the signal peptide of the protein OmpA from *E. coli* followed by the periplasmic domain of CbcL and a C-terminal Strep-tag^®^.

### Protein Expression and Purification

Isolated colonies of *E. coli* Tuner (DE3)/pEC86+pVA203-CbcL-St were selected and inoculated in liquid 2xYT medium supplemented with 100 μg⋅mL^–1^ of ampicillin and 34 μg⋅mL^–1^ of chloramphenicol, and incubated overnight at 30°C and 200 rpm. On the following day, 15 mL of this culture were transferred to 2 L conical flasks, each containing 1 L of liquid 2xYT medium with antibiotics in the same concentration. The cultures were incubated at 30°C and 180 rpm for approximately 8 h until an OD_600_ ∼1.7 was reached. Cells were collected by centrifugation at 5,500 *×g* for 15 min at 4°C. The pellets were then resuspended in buffer W (100 mM Tris-HCl pH 8, 150 mM NaCl, 1mM EDTA), in a ratio of 1 mL of buffer per gram of cells and frozen at −20°C. Cell lysis was performed by a combined method of three freeze/thaw cycles followed by 18 cycles of ultrasonication (3 min on plus 1 min off) with an Ultrasonic homogenizer (Branson) regulated for 65% of amplitude and in the presence of DNase I, 2 mM benzamidine and 1 mM phenylmethanesulfonylfluoride. Cell debris were removed by centrifugation at 48,000 *×g* for 1 h at 6°C. The supernatant containing the soluble extract was loaded directly onto a 5 mL Strep-Tactin^®^ XT 4Flow (IBA Lifesciences) column, previously equilibrated with buffer W. The flowthrough was collected and the unbound proteins washed by passing 25 mL of buffer W. The protein was eluted with buffer BXT (buffer W with 50 mM biotin). The eluted sample was dialyzed against 10 mM Tris-HCl pH 8, using a dialysis membrane with a molecular weight cut off (MWCO) 12–14 kDa (Spectrum). After the dialysis step, the sample was loaded onto a 5 mL anion exchange UNOsphere™ Q (Bio-Rad) column, equilibrated with the same dialysis buffer. The protein was eluted with a 75 mL gradient of 0–300 mM NaCl. The fractions were analyzed by SDS-PAGE (12.5%) and stained with BlueSafe (NZYTech). Fractions containing CbcL (unless stated otherwise CbcL’s periplasmatic domain will be referred as CbcL) were concentrated and the buffer exchanged using an Amicon Ultra-4 centrifugal filter unit with a MWCO 10 kDa (Merck-Millipore). After, the samples were loaded onto a Superdex™ 75 Increase 10/300 GL (GE Healthcare) equilibrated with 100 mM sodium phosphate buffer pH 8. Chromatographic steps were performed with an ÄKTA Pure or with an ÄKTA Prime Plus. Final protein purity was evaluated by SDS-PAGE. The molecular weight of CbcL was confirmed by matrix assisted laser desorption ionization coupled to time-of-flight mass spectrometry (MALDI-TOF/TOF MS) performed by the Mass Spectrometry Unit (UniMS), ITQB/iBET, Oeiras, Portugal. For interaction studies with CbcL, PpcA was expressed and purified as previously described (Londer et al., 2002).

### UV-Visible Spectroscopy, Protein Quantification, Heme Content and Molar Extinction Coefficient Determination

UV-visible spectra were acquired on a Thermo Scientific Evolution 201 spectrophotometer at room temperature in the reduced and oxidized states. A freshly prepared solution of sodium dithionite was added to the sample to reduce the protein. Protein concentration was measured with the Pierce™ Modified Lowry Protein Assay Kit (Thermo Scientific Pierce; Ohnishi and Barr, 1978), using horse heart cytochrome *c* as protein standard, and used to determine molar extinction coefficients. Subsequent protein concentrations were calculated using the determined molar extinction coefficients. The protein heme content was determined by the pyridine hemochromogen assay, by measuring the absorbance at 550 nm (for *c*-type hemes ϵ_550nm_ = 30.27 mM^–1^⋅cm^–1^) of a 1.2 μM CbcL solution prepared in 50 mM of NaOH/20% pyridine and reduced by the addition of a freshly prepared sodium dithionite solution (Berry and Trumpower, 1987).

### Circular Dichroism Spectroscopy

CD characterization in the Far-UV was performed with a CbcL sample at 10 μM prepared in 10 mM sodium phosphate buffer pH 8. Measurements were performed in an Applied Photophysics Chirascan™ qCD spectropolarimeter using a 300 μL quartz cuvette with 1 mm of path length. The CD spectra at 25°C are an average of three spectral acquisitions with a step-size of 1 nm. A temperature ramp was measured in the range of 10–94°C, with a temperature increment of 2°C for each measurement with 1 min period for stabilization in each temperature. A two-state transition model from folded to unfolded was used to determine the melting temperature and the enthalpy of unfolding (ΔH) (Greenfield, 2007b). The analysis of the CD spectra, including the determination of the composition of the secondary structure elements, was carried out using the online program DichroWeb with the CDSSTR algorithm and SPM180 as a reference dataset (Whitmore and Wallace, 2004).

### Nuclear Magnetic Resonance Spectroscopy

NMR experiments were performed either on a Bruker Avance Neo 500 MHz spectrometer or on a Bruker Avance III 600 MHz spectrometer at 25°C. The water signal was used as internal reference for the calibration of the ^1^H chemical shifts to sodium trimethylsilylpropanesulfonate (Pierattelli et al., 1996). All 1D ^1^H NMR spectra were acquired with 1024 increments, a sweep width of 70 kHz and processed using TOPSPIN 4.1.1™ (Bruker).

#### Electron Transfer Experiments

To monitor the electron transfer reaction between CbcL and PpcA, a sample of 100 μM (160 μL) of CbcL prepared in 10 mM sodium phosphate buffer pH 8 was lyophilized (NMR spectra were acquired before and after lyophilization − in the reduced and oxidized states − to confirm that the protein integrity and their redox behavior was not affected) and resuspended in ^2^H_2_O inside an anaerobic glovebox (LABstar, MBraun) to avoid the presence of oxygen. The NMR tube was sealed with a gas-tight serum cap and flushed with gaseous hydrogen for reduction in the presence of a catalytic amount of hydrogenase from *Desulfovibrio vulgaris*. After reduction, argon was used to remove all the hydrogen from the sample. Similarly, a sample of PpcA was lyophilized and resuspended in 20 μL of ^2^H_2_O inside the glovebox, resulting in a 3.2 mM sample. The electron transfer reaction between reduced CbcL and oxidized PpcA was followed by a series of 1D ^1^H NMR spectra each acquired after the addition of increasing equivalents of PpcA to the reduced CbcL sample, inside the anaerobic glovebox.

#### Protein-Protein Interaction Studies in the Oxidized Form

The molecular interactions between CbcL and PpcA were followed by NMR chemical shift perturbation experiments. For the monitorization of perturbations on PpcA’s NMR signals, a sample of 50 μM PpcA (160 μL) and a sample of CbcL at 800 μM (30 μL) both in 10 mM sodium phosphate buffer pH 8 were prepared by lyophilization and resuspended in ^2^H_2_O. A series of 1D ^1^H NMR spectra were obtained after the addition of increasing amounts of CbcL to PpcA. For the monitorization of perturbations on CbcL’s NMR signals, a sample of 100 μM CbcL (160 μL) and a sample of PpcA at 3.2 mM (50 μL) both in 10 mM sodium phosphate buffer pH 8 were lyophilized and resuspended in ^2^H_2_O. A series of 1D ^1^H NMR spectra were obtained after the addition of increasing amounts of PpcA to CbcL. The pH was monitored to confirm that it was maintained throughout the experiments.

The chemical shift variations of the heme methyl signals of PpcA were used to determine the dissociation constant (*K*_d_). The *K*_d_ was determined by a two-parameter non-linear least-square fitting estimated under fast exchange conditions for a one binding site model corrected for the dilution effect, using OriginPro 8.5. The value was determined by the following equation, as described by Kannt et al. (1996).


$$\Delta \,\delta_{\text{bind}}=0.5\,\Delta \,\delta_{\max}\,\left(\text{A}-\sqrt{{\text{A}}^{2}-4\,\text{R}}\right),$$

$$\mathrm{with}\,A=1+R+K_{d}\,\frac{[{\text{CbcL]}}_{0}+{\text{R[PpcA]}}_{0}}{[{\text{CbcL]}}_{0}{\text{[PpcA]}}_{0}}$$

In the equation, Δδ_bind_ is the chemical shift change at a determined protein ratio, Δδ_max_ is the maximum chemical shift difference between the free and complex form of PpcA, R is the [CbcL]/[PpcA] ratio at each point. [PpcA]_0_ and [CbcL]_0_ correspond to the stock concentrations of each protein.

### Redox Titrations Monitored by UV-Visible Spectroscopy

Redox titrations were performed in anaerobic conditions with ∼3 μM of CbcL in 80 mM sodium phosphate buffer with NaCl (250 mM final ionic strength) pH 8. The UV-visible spectrophotometer (Thermo Scientific Evolution 300) was coupled to a circulating bath to keep the sample at 15°C. The solution potential was measured by a platinum pin electrode associated with an AgCl/Ag reference (Crison) and corrected for the Standard Hydrogen Electrode (SHE). The electrode was calibrated at the start and at the end of each titration with freshly prepared saturated quinhydrone solutions at pH 7 and pH 4. The following mixture of redox mediators, each at 1 μM final concentration, was added to the protein solution to promote a fast exchange between the redox centers of the protein and the electrode: 1,2-naphtoquinone-4-sulphonic acid (E^0^′ = +215 mV), 1,2-napthoquinone (E^0^′ = +143 mV), trimethylhydroquinone (E^0^′ = +115 mV), phenazine methosulfate (E^0^′ = +80 mV), phenazine ethosulfate (E^0^′ = +55 mV), gallocyanine (E^0^′ = +21 mV), methylene blue (E^0^′ = +11 mV), indigo tetrasulfonate (E^0^′ = −30 mV), indigo trisulfonate (E^0^′ = −70 mV), indigo disulfonate (E^0^′ = −120 mV), 2-hidroxy-1,4-naphthoquinone (E^0^′ = −145 mV), anthraquinone-2,6-disulfonate (E^0^′ = −185 mV), anthraquinone-2-sulfonate (E^0^′ = −225 mV), safranine O (E^0^′ = −280 mV), neutral red (E^0^′ = −325 mV), benzyl viologen (E^0^′ = −345 mV), diquat (E^0^′ = −350 mV) and methyl viologen (E^0^′ = −440 mV) (Dutton, 1978). Potassium ferricyanide and sodium dithionite were used as oxidizing and reducing agents, respectively. The experiment was performed two times, and the reduction potentials were found to be reproducible within ± 2 mV.

The reduced fraction of CbcL was calculated by integrating the area of the α band above the isosbestic points (541 and 559 nm), as previously described (Paquete et al., 2007). The macroscopic apparent midpoint reduction potential (*E*_*app*_) value was determined after a nonlinear fit of the experimental data to a model with nine sequential one electron Nernst equations using OriginPro 8.5.

## Results and Discussion

### Production of CbcL

**FIGURE 1:**
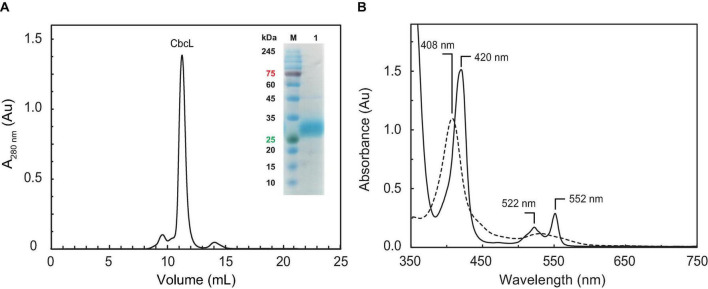
Purification of cytochrome CbcL and its UV-visible spectral features. **(A)** Size exclusion chromatography elution profile for the last step of CbcL purification. The inset shows an SDS-PAGE gel for purified CbcL (lane 1) and molecular weight marker (lane M). **(B)** UV-visible spectra of CbcL (1 **μ**M in 10 mM phosphate buffer pH 8) at room temperature. The solid and dashed lines correspond to the reduced and oxidized state (as purified), respectively.

The purification of CbcL involved three consecutive chromatographic steps: an affinity step followed by an anion exchange and a size exclusion step (Figure 1A). The purity of CbcL was evaluated by SDS-PAGE (Figure 1A inset) and confirmed by MALDI-TOF/TOF-MS. The peak for pure CbcL in the mass spectra at 34.5 kDa is in agreement with the expected molecular mass of 28.9 kDa of the apo-protein plus 5.6 kDa for the nine heme groups. The presence of the nine heme groups was further confirmed by the pyridine hemochrome assay. The molar extinction coefficients for CbcL were determined by the Lowry colorimetric assay at 408 nm in the oxidized form (ε_408nm_ = 923 mM^–1^⋅cm^–1^) and at 552 nm in the reduced form (ε_552nm_ = 230 mM^–1^⋅cm^–1^) and were used to calculate the purification yield of CbcL per liter of culture (0.3 mg).

### Analysis of CbcL Amino Acid Sequence and Structure Prediction

The local alignment search tool (BLAST) was used to search for similar amino acid sequences to full-length CbcL in the database from NCBI (Altschul et al., 1997). The alignment of the sequences with more than 65% pairwise identity was performed with Clustal Omega (Supplementary Figure 1) (Sievers and Higgins, 2018). The sequences correspond to proteins from the Desulfuromonadales order belonging to Deltaproteobacteria in which the nine binding motifs (CXXCH) characteristic of *c*-type hemes are conserved. The alignment between CbcL and homologous sequences shows ten conserved histidine residues in the periplasmic domain, in addition to those of the binding motifs, and no conserved methionine residues, suggesting that all the hemes are bis-histidine coordinated. This was further confirmed by the AlphaFold protein structure prediction method (Jumper et al., 2021) using the ChimeraX software tool (Pettersen et al., 2021) (Supplementary Figure 2).

### Spectroscopic Characterization of CbcL

**FIGURE 2:**
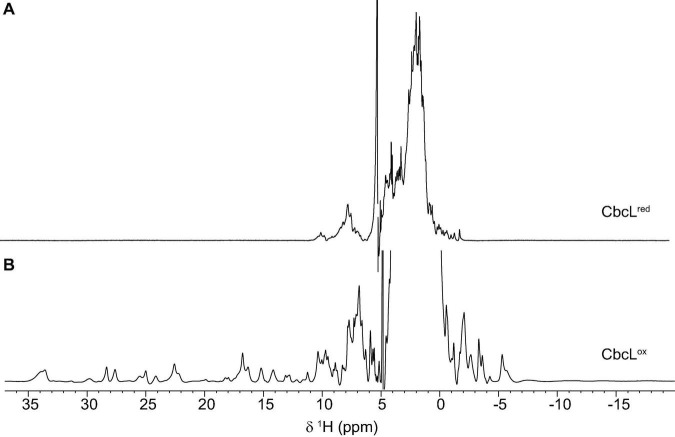
1D ^1^H NMR spectra of cytochrome CbcL in the reduced **(A)** and oxidized **(B)** forms. The spectra were acquired at 25°C with 100 μM of CbcL in 10 mM sodium phosphate pH 8.

Complementary spectroscopic techniques were used for the structural and functional characterization of CbcL. The UV-visible spectra of the oxidized and reduced states (Figure 1B) showed features characteristic of low-spin hexacoordinated *c*-type hemes: the Soret band (at 408 and 420 nm in the oxidized and reduced states, respectively), and the typical β and α bands at 522 and 552 nm in the reduced state. The spin state of the heme groups was further confirmed by NMR. The 1D ^1^H NMR spectra of cytochromes provide important information regarding the spin-state of the heme groups and their axial ligands. In fact, the signals for high- or low-spin hemes appear in very distinct spectral regions for each redox state. Cytochromes containing high-spin hemes typically show broad heme methyl signals above 40 ppm (Moore and Pettigrew, 1990). On the other hand, for low-spin hemes these resonances are mainly found up to 35 ppm. For the reduced form, the spectra are also distinct. Cytochromes containing high-spin hemes show larger spectral regions, typically from 30 to −15 ppm, compared to those with low-spin hemes (from 10 to −5 ppm). In the case of CbcL, the 1D ^1^H NMR spectra in the oxidized and reduced states are considerably different (Figure 2). In the reduced state (Figure 2A), the signals cover the spectral width between 10 and −5 ppm, which is characteristic of a low spin cytochrome (Fe(II), S = 0). In the oxidized state, the signals are broader and cover a larger spectral region from 35 to −7 ppm, as expected from the paramagnetic effect caused by the unpaired electron of each heme (Figure 2B). The shape and the spectral region covered by the signals in the oxidized state also confirms that the heme groups are low-spin (Fe(III), S = ½).

**FIGURE 3:**
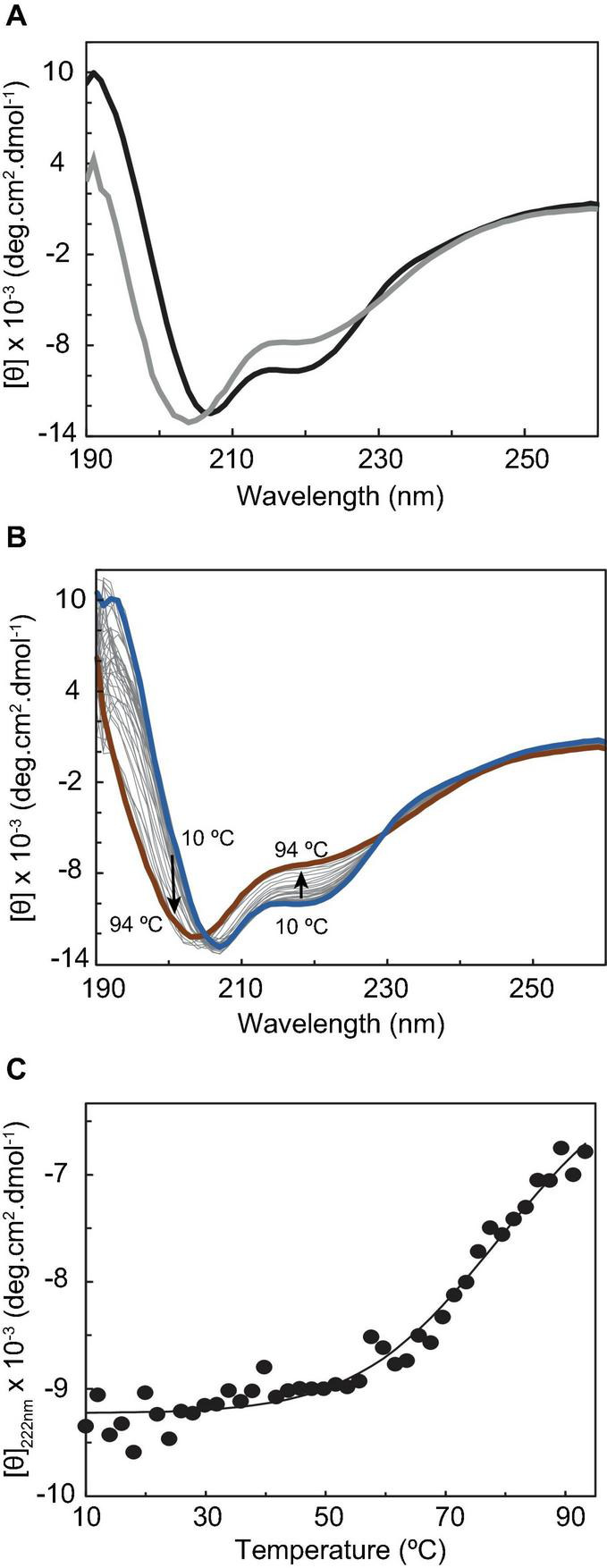
Circular dichroism studies of cytochrome CbcL. **(A)** Far-UV CD spectra of CbcL (10 μM in 10 mM sodium phosphate pH 8) as purified (black) and after the temperature ramp (gray) at 25°C. **(B)** Far-UV CD spectra of CbcL at different temperatures (10–94°C). **(C)** Thermal unfolding of CbcL. Mean residue ellipticity [θ] variation at 222 nm as a function of temperature. The solid line represents the fitting of the experimental data to a two-state transition, resulting in a melting temperature of 81 ± 5°C.

Circular dichroism (CD) spectroscopy in the far-UV region (190–260 nm) was then used to probe the secondary structural elements and the thermal stability of CbcL. The CD spectrum of CbcL (Figure 3A, black line) has a positive maximum at 191 nm and two minima at 207 nm and 218. The signal at 207 is characteristic of α-helical structures whereas that at 218 nm represents a mixture of α-helix and β-sheet structure (Wei et al., 2014). The percentages of secondary structural elements of CbcL were calculated by the program DichroWeb (Whitmore and Wallace, 2004) and are listed in Table 1. The results obtained show that the secondary structure of CbcL is mostly disordered (37%) followed by 29, 18, and 15% of α-helix, β-sheet and turns, respectively. As observed for other multiheme cytochromes, CbcL also has a low ratio of amino acids per heme (28 residues), which restrains the amount of ordered secondary structure as observed for example for the triheme cytochrome PpcA (Pokkuluri et al., 2011) and the dodecaheme cytochrome GSU1996 (Pokkuluri et al., 2010), which have 24 and 26 residues per heme, respectively (Table 1). This is also in agreement with the structural model predicted by AlphaFold that mostly shows disordered and helical elements (Supplementary Figure 2).

**TABLE 1:** Structural features of cytochromes CbcL, PpcA and GSU1996 from *G. sulfurreducens*.

Cytochromes	Secondary structure elements (%)				Number of residues	Number of heme groups
	
	α-Helix	β-Sheet	Turn	Disordered		
CbcL (25°C)^1^	29	18	15	37	250	9
PpcA^2^	28	15	41	17	71	3
GSU1996^2^	19	12	52	18	318	12

*The percentage of secondary structure elements of CbcL was determined by CD spectroscopy in the far-UV region. Data for PpcA and GSU1996 is also presented for comparison.*

*^1^According to the results obtained from the DichroWeb online platform.*

*^2^According to the results obtained from the online platform STRIDE (Frishman and Argos, 1995) using the PDB files: 2MZ9 (Pokkuluri et al., 2004b) and 3OV0 (Pokkuluri et al., 2011) for PpcA and GSU1996, respectively.*

The conformational stability of CbcL was assessed by performing temperature-induced denaturation (from 10 to 94°C), monitored by far-UV CD at 222 nm, which reports on the stability of the α-helical secondary structural elements. The data show that the protein unfolding results in the loss of secondary structure, evidenced by the decrease in the ellipticity of the 222 nm signal, particularly above 50°C (Figures 3B,C). An unfolding enthalpy of 82.0 ± 13.2 kJ⋅mol^–1^ (19.6 ± 3.1 kcal⋅mol^–1^) was also determined. This value is in line with enthalpy values of unfolding for model monomer proteins (17.7 and 21.5 kcal⋅mol^–1^) (Greenfield, 2007a). Analyzing the spectra before and after the temperature ramp, it can also be observed that the thermal unfolding is not fully reversible, as the far-UV CD absorption fingerprints are not fully restored. In fact, after the temperature ramp, the spectrum obtained at 25°C (Figure 3A, gray line) shows a shift of the negative band maximum from 207 to 204 nm, as well as a considerable change in ellipticity at 191 nm, when compared with the spectrum obtained before the temperature ramp.

### Functional Characterization of CbcL

**FIGURE 4:**
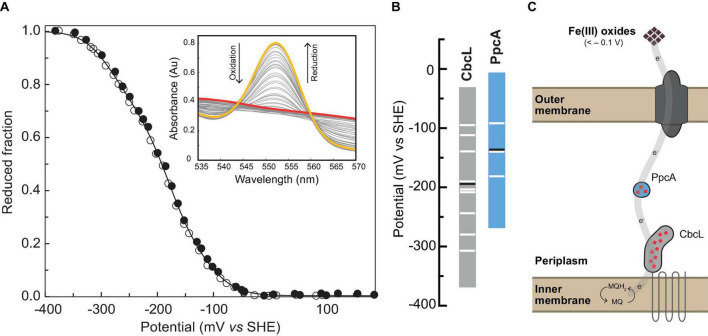
Redox characterization of cytochrome CbcL. **(A)** Redox titrations at pH 8 monitored by UV-visible spectroscopy. The experimental data are represented by closed (reductive direction) and open (oxidative direction) circles. The solid line represents the fitted curve. The inset shows the α-band region monitored throughout the redox titration. Fully reduced and fully oxidized spectra are represented in yellow and red, respectively. **(B)** Redox-active windows (1–99% range for protein reduction/oxidation) of CbcL (this work) and PpcA (Morgado et al., 2008) at pH 8. Horizontal black lines correspond to the *E*_*app*_ of each cytochrome, and horizontal white lines correspond to the fitted sequential macroscopic reduction potential values. **(C)** Proposed model for extracellular electron transfer to Fe(III) oxides and electrodes poised at -100 mV in *G. sulfurreducens*.

The next step on the characterization of CbcL was the determination of its reduction potential and redox working functional range. The distinct spectral UV-visible spectroscopic features of CbcL in the oxidized and reduced forms (Figure 1B) were explored to monitor the variation of its reduced fraction with the solution redox potential. Thus, redox titrations followed by UV-visible spectroscopy were performed for CbcL at pH 8 by monitoring the variation of the α-band (552 nm) with the solution redox potential (Figure 4A). The apparent reduction potential (*E*_*app*_) of −194 ± 2 mV was determined for CbcL. Given the cellular localization of CbcL at the inner membrane, it is most likely that the electrons are transferred from CbcL to periplasmic proteins also involved in the same extracellular electron transfer pathway. The triheme cytochrome PpcA is the most abundant periplasmic cytochrome and is a putative redox partner of CbcL. The *E*_*app*_ value of PpcA (−138mV (Morgado et al., 2008)) is less negative then the one obtained for CbcL, suggesting a thermodynamically favorable electron transfer from CbcL to PpcA.

### Monitorization of the Electron Transfer Reaction Between CbcL and PpcA by NMR

**FIGURE 5:**
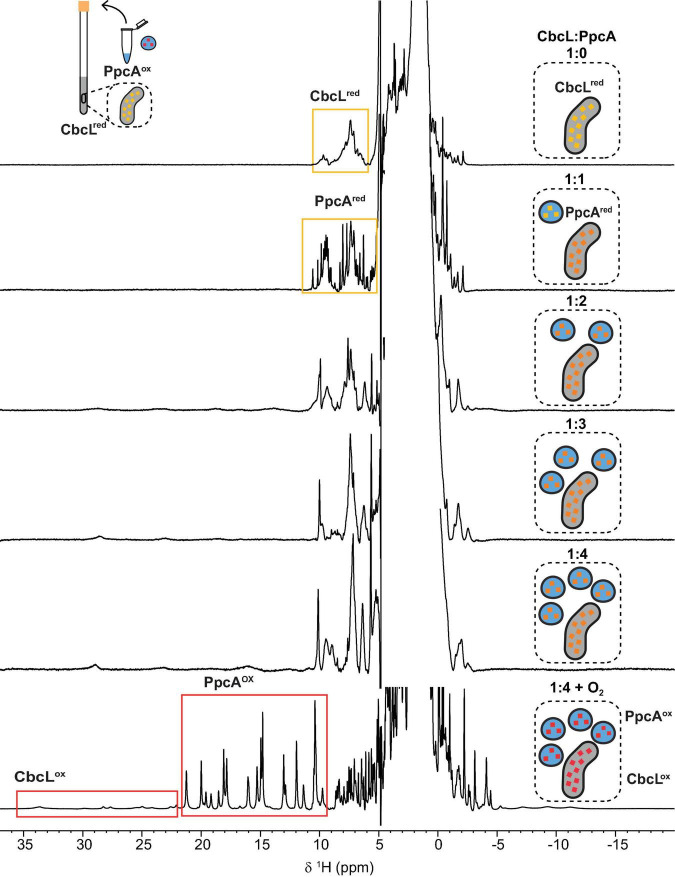
Monitorization of the electron transfer reaction between cytochromes CbcL and PpcA by NMR. 1D ^1^H NMR spectra were acquired at 25°C with 100 μM of CbcL in 10 mM sodium phosphate pH 8 with increasing equimolar amounts of PpcA, up to 1:4 ratio. The rectangles highlight the cytochromes’ fingerprints in the reduced and oxidized states. The schemes represent the addition of equimolar amounts of oxidized PpcA (blue) to the NMR tube containing initially reduced CbcL (yellow). Red represents fully oxidized states, yellow fully reduced states, and intermediate redox states are represented in orange.

The redox properties of CbcL and PpcA, as well as their cellular localization, strongly suggest that CbcL is most likely able to transfer electrons to PpcA. To verify this hypothesis, the electron transfer reaction between CbcL and PpcA was probed by NMR. The spectral features of the 1D ^1^H NMR spectra of CbcL and PpcA in the reduced and oxidized states were used to assess the electron transfer reaction between the two proteins, following a newly developed strategy to monitor electron transfer between cytochromes (Morgado and Salgueiro, 2022). Thus, a reduced sample of CbcL (CbcL^red^) was prepared and then titrated with increasing equimolar amounts of oxidized PpcA (PpcA^ox^). After the first addition of PpcA^ox^ (Figure 5, 1:1 NMR spectrum) the signals characteristic of reduced PpcA (PpcA^red^; Supplementary Figure 3) are visible in the region between 5 and 11 ppm (yellow rectangle in Figure 5) and no typical fingerprint of PpcA^ox^ is observed (resonances between 11 and 22 ppm), confirming that electrons were transferred from CbcL to PpcA.

Following the subsequent additions of PpcA^ox^ the NMR signals of CbcL in the low field region of the spectra above 22 ppm are visible. Since the reduction potential of CbcL is more negative and it has three times more hemes than PpcA, it could be expected that, after the addition of three molar equivalents of PpcA^ox^, CbcL would be fully oxidized. However, this was not observed and, instead, CbcL and PpcA were both in intermediate oxidation states since broad signals are observed between 11 and 30 ppm (1:3 NMR spectrum in Figure 5). Even after the addition of four molar equivalents of PpcA^ox^, an intermediate oxidation state remained for both cytochromes (1:4 NMR spectrum in Figure 5). To confirm that the two cytochromes were in an intermediate oxidation state, the NMR tube was unsealed. The contact with atmospheric O_2_ led to the fully oxidation of both cytochromes, as confirmed by the typical signals of CbcL^ox^ and PpcA^ox^ (1:4 + O_2_ NMR spectrum in Figure 5). This experimental setup allowed to observe the partial reduction of PpcA with electrons provided by CbcL, indicating that this redox pair is indeed able to transfer electrons with the directionality expected from their reduction potential values. The results also indicate that the physiological state of the cytochromes is neither fully reduced nor oxidized. Indeed, the difference of 56 mV in their apparent midpoint reduction potential values assures an overlap of their redox windows (Figure 4B). This overlap explains why electrons are not fully transferred from CbcL to PpcA. Instead, they remain in equilibrium acting as a reservoir of electrons to permit a constant flow whenever CbcL is loaded with electrons by the quinone pool, and PpcA oxidized by its redox partner (Figure 4C).

### Interaction Studies Between CbcL and the Periplasmic Cytochrome PpcA

**FIGURE 6:**
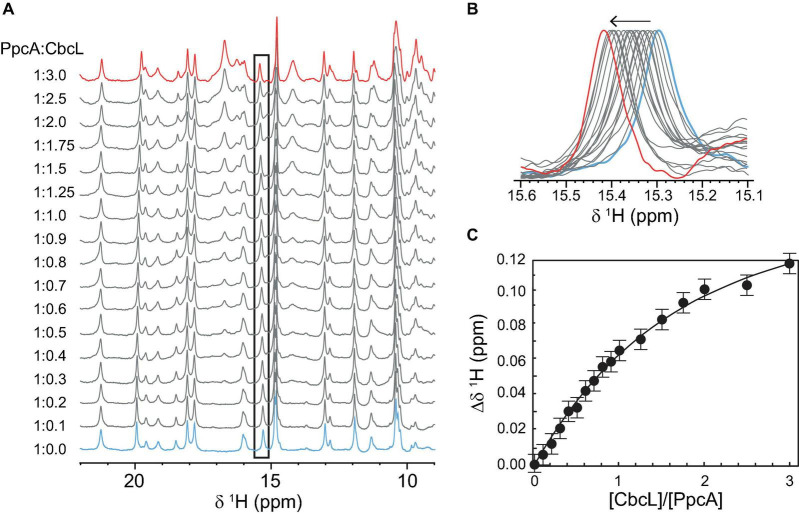
Binding studies between cytochromes PpcA and CbcL followed by NMR chemical shift perturbation experiments. **(A)** 1D ^1^H NMR spectra of PpcA acquired with increasing amounts of CbcL (ratio PpcA:CbcL indicated on the left side of each spectrum). Spectra were acquired at 25°C with 50 μM of PpcA in 10 mM sodium phosphate pH 8. **(B)** Expansion of the region containing the most affected heme methyl signal 18^1^CH_3_^I^. **(C)** Binding curve for CbcL and PpcA monitored by the chemical shift changes of heme methyl 18^1^CH_3_^I^. The error bars represent estimated experimental errors. The solid line represents the fitting for a *K*_d_ of 57 ± 9 μM.

Having shown that CbcL and PpcA can exchange electrons, the distinct NMR spectral features of the two cytochromes were then explored to determine the affinity constant of the redox complex. NMR chemical shift perturbation experiments have been used to probe interacting regions between redox proteins, particularly in the oxidized state for which the signal dispersion is considerably larger compared to the reduced form (Bashir et al., 2011; Fonseca et al., 2013; Dantas et al., 2017; Fernandes et al., 2017). This is case of cytochromes CbcL and PpcA whose spectra in the oxidized state are considerably different (Figure 2). In the case of redox complexes between multiheme *c*-type cytochromes, it is expected that at least one heme from each protein would be in close contact. Thus, if interacting, the chemical environment in the vicinity of these groups would be altered and the chemical shift (or broadening) of the heme methyl(s) signal(s) would be affected. Therefore, in the present work, NMR chemical shift perturbation experiments were carried out for the two cytochromes in the oxidized state by adding successive amounts of one cytochrome to the other. Supplementary Figure 4 depicts the 1D ^1^H NMR spectra of CbcL in the low-field region titrated with increasing amounts of PpcA. The heme methyl signals of CbcL that are detected between 22 and 35 ppm are not affected upon addiction of PpcA. However, this does not completely exclude the interaction, since the binding region could be in the vicinity of the heme groups whose resonances are not detected in that region of the spectra. Consequently, the chemical shifts perturbations were also probed for PpcA heme methyl signals (Figure 6A). The higher perturbation was observed for the heme methyl 18^1^CH_3_^I^ and the variation of its chemical shift was used to determine the dissociation constant (*K*_d_) of the complex (Figures 6B,C). The value obtained in the micromolar range (57 ± 9 μM) suggests the formation of a low affinity complex characteristic of redox partners and is in line with values previously reported for redox proteins (Bashir et al., 2011; Meschi et al., 2011; Fonseca et al., 2013).

## Conclusion

Multiheme cytochromes have a crucial role in extracellular electron transfer mechanisms in exoelectrogenic microorganisms, acting as electron capacitors and carriers. Cytochrome CbcL is one of the inner membrane quinone oxidoreductases identified in *G. sulfurreducens* and was shown to be essential for the reduction of extracellular electron acceptors with reduction potentials lower than −100 mV. CbcL is composed by a transmembrane domain and a soluble periplasmic domain, and the latter is a *c*-type cytochrome with nine heme groups and was biochemically characterized in this work. The spectroscopic characterization revealed that the hemes are low spin and coordinated by two histidine residues and that the polypeptide chain has a low content of secondary structural elements, features that are typically observed for multiheme cytochromes. Compared to the one of the periplasmic cytochrome PpcA (−138 mV), the reduction potential value of CbcL (−194 mV) is more negative. This suggests a thermodynamically favorable electron transfer from CbcL to PpcA and a putative formation of a redox complex. Using NMR it was possible to unequivocally confirm the formation of this complex. In fact, direct electron transfer from reduced CbcL to oxidized PpcA was observed in 1D ^1^H NMR titrations. Additionally, NMR chemical shift perturbation experiments also showed the formation of a low affinity complex between the two cytochromes.

The unequivocal demonstration of the formation of a complex between CbcL and PpcA, as well as the superimposition of their redox active windows assures the continuous flow of electrons from the inner membrane towards the periplasm and ultimately to the extracellular electron acceptors.

## Data Availability Statement

The original contributions presented in the study are included in the article/Supplementary Material, further inquiries can be directed to the corresponding authors.

## Author Contributions

LM and CS designed and supervised the project. JA, MS, and LM performed the experiments and data analysis. JA, CS, and LM wrote the manuscript. All authors contributed to the article and approved the submitted version.

## Conflict of Interest

The authors declare that the research was conducted in the absence of any commercial or financial relationships that could be construed as a potential conflict of interest.

## Publisher’s Note

All claims expressed in this article are solely those of the authors and do not necessarily represent those of their affiliated organizations, or those of the publisher, the editors and the reviewers. Any product that may be evaluated in this article, or claim that may be made by its manufacturer, is not guaranteed or endorsed by the publisher.

## References

[B1] Al-Amshawee S. Yunus M. Y. B. M. Azoddein A. A. M. Hassell D. G. Dakhil I. H. Hasan H. A. (2020). Electrodialysis desalination for water and wastewater: a review. *Chem. Eng. J.* 380:122231. 10.1016/j.cej.2019.122231

[B2] Altschul S. F. Madden T. L. Schäffer A. A. Zhang J. Zhang Z. Miller W. (1997). Gapped BLAST and PSI-BLAST: a new generation of protein database search programs. *Nucleic Acids Res.* 25 3389–3402. 10.1093/nar/25.17.3389 9254694 PMC146917

[B3] Bashir Q. Scanu S. Ubbink M. (2011). Dynamics in electron transfer protein complexes. *FEBS J.* 278 1391–1400. 10.1111/j.1742-4658.2011.08062.x 21352493

[B4] Berry E. A. Trumpower B. L. (1987). Simultaneous determination of hemes *a*, *b*, and *c* from pyridine hemochrome spectra. *Anal. Biochem.* 161 1–15. 10.1016/0003-2697(87)90643-9 3578775

[B5] Cavalier-Smith T. Brasier M. Embley T. M. (2006). Introduction: how and when did microbes change the world? *Philos. Trans. R. Soc. B Biol. Sci.* 361 845–850. 10.1098/rstb.2006.1847 16754602 PMC1626534

[B6] Dantas J. M. Brausemann A. Einsle O. Salgueiro C. A. (2017). NMR studies of the interaction between inner membrane-associated and periplasmic cytochromes from *Geobacter sulfurreducens*. *FEBS Lett.* 591 1657–1666. 10.1002/1873-3468.12695 28542725

[B7] Fernandes A. P. Nunes T. C. Paquete C. M. Salgueiro C. A. (2017). Interaction studies between periplasmic cytochromes provide insights into extracellular electron transfer pathways of *Geobacter sulfurreducens*. *Biochem. J.* 474 797–808. 10.1042/BCJ20161022 28093471

[B8] Fonseca B. M. Paquete C. M. Neto S. E. Pacheco I. Soares C. M. Louro R. O. (2013). Mind the gap: cytochrome interactions reveal electron pathways across the periplasm of *Shewanella oneidensis* MR-1. *Biochem. J.* 449 101–108. 10.1042/BJ20121467 23067389

[B9] Frishman D. Argos P. (1995). Knowledge-based secondary structure assignment. *Proteins Struct. Funct. Genet.* 23 566–579. 10.1093/nar/gkh429 8749853

[B10] Greenfield N. J. (2007b). Using circular dichroism spectra to estimate protein secondary structure. *Nat. Protoc.* 1 2876–2890. 10.1038/nprot.2006.202 17406547 PMC2728378

[B11] Greenfield N. J. (2007a). Using circular dichroism collected as a function of temperature to determine the thermodynamics of protein unfolding and binding interactions. *Nat. Protoc.* 1 2527–2535. 10.1038/nprot.2006.204 17406506 PMC2752288

[B12] Joshi K. Chan C. H. Bond D. R. (2021). *Geobacter sulfurreducens* inner membrane cytochrome CbcBA controls electron transfer and growth yield near the energetic limit of respiration. *Mol. Microbiol.* 116 1124–1139. 10.1111/mmi.14801 34423503

[B13] Jumper J. Evans R. Pritzel A. Green T. Figurnov M. Ronneberger O. (2021). Highly accurate protein structure prediction with AlphaFold. *Nature* 596 583–589. 10.1038/s41586-021-03819-2 34265844 PMC8371605

[B14] Kanehisa M. Sato Y. Kawashima M. Furumichi M. Tanabe M. (2016). KEGG as a reference resource for gene and protein annotation. *Nucleic Acids Res.* 44 D457–D462. 10.1093/nar/gkv1070 26476454 PMC4702792

[B15] Kannt A. Young S. Bendall D. S. (1996). The role of acidic residues of plastocyanin in its interaction with cytochrome *f*. *Biochim. Biophys. Acta Bioenerg.* 1277 115–126. 10.1016/S0005-2728(96)00090-4 30897681

[B16] Koffi N. J. Okabe S. (2020). High voltage generation from wastewater by microbial fuel cells equipped with a newly designed low voltage booster multiplier (LVBM). *Sci. Rep.* 10:18985. 10.1038/S41598-020-75916-7 33149238 PMC7642417

[B17] Kumar R. Singh L. Wahid Z. A. Din M. F. M. (2015). Exoelectrogens in microbial fuel cells toward bioelectricity generation: a review. *Int. J. Energy Res.* 39 1048–1067. 10.1002/er.3305

[B18] Dutton P. L. (1978). Redox potentiometry: determination of midpoint potentials of oxidation-reduction components of biological electron-transfer systems. *Methods Enzymol.* 54 411–435. 10.1016/S0076-6879(78)54026-3 732578

[B19] Levar C. E. Chan C. H. Mehta-Kolte M. G. Bond D. R. (2014). An inner membrane cytochrome required only for reduction of high redox potential extracellular electron acceptors. *MBio* 5:e0203414. 10.1128/mBio.02034-14 25425235 PMC4251993

[B20] Logan B. E. Rossi R. Ragab A. Saikaly P. E. (2019). Electroactive microorganisms in bioelectrochemical systems. *Nat. Rev. Microbiol.* 17 307–319. 10.1038/s41579-019-0173-x 30846876

[B21] Londer Y. Y. Pokkuluri P. R. Tiede D. M. Schiffer M. (2002). Production and preliminary characterization of a recombinant triheme cytochrome *c*_7_ from *Geobacter sulfurreducens* in *Escherichia coli*. *Biochim. Biophys. Acta - Bioenerg.* 1554 202–211. 10.1016/S0005-2728(02)00244-X 12160993

[B22] Lovley D. R. Holmes D. E. Nevin K. P. (2004). Dissimilatory Fe(III) and Mn(IV) reduction. *Adv. Microb. Physiol.* 49 219–286. 10.1016/S0065-2911(04)49005-5 15518832

[B23] Lovley D. R. Ueki T. Zhang T. Malvankar N. S. Shrestha P. M. Flanagan K. A. (2011). Geobacter. The Microbe Electric’s Physiology, Ecology, and Practical Applications. *Adv Microb Physiol* 59 1–100. 10.1016/B978-0-12-387661-4.00004-5 22114840

[B24] Marsili E. Sun J. Bond D. R. (2010). Voltammetry and growth physiology of *Geobacter sulfurreducens* biofilms as a function of growth stage and imposed electrode potential. *Electroanalysis* 22 865–874. 10.1002/elan.200800007

[B25] Meschi F. Wiertz F. Klauss L. Blok A. Ludwig B. Merli A. (2011). Efficient electron transfer in a protein network lacking specific interactions. *J. Am. Chem. Soc.* 133 16861–16867. 10.1021/ja205043f 21916462

[B26] Moore G. W. Pettigrew G. R. (1990). *Cytochromes c: Evolutionary, Structural and Physicochemical Aspects.* New York: Springer Science & Business Media.

[B27] Morgado L. Bruix M. Orshonsky V. Londer Y. Y. Duke N. E. C. Yang X. (2008). Structural insights into the modulation of the redox properties of two *Geobacter sulfurreducens* homologous triheme cytochromes. *Biochim. Biophys. Acta - Bioenerg.* 1777 1157–1165. 10.1016/j.bbabio.2008.04.043 18534185

[B28] Morgado L. Salgueiro C. A. (2022). Elucidation of complex respiratory chains: a straightforward strategy to monitor electron transfer between cytochromes. *Metallomics* mfac012. [Epub online ahead of print]. 10.1093/mtomcs/mfac012 35225346

[B29] Nealson K. H. Saffarini D. (1994). Iron and manganese in anaerobic respiration: environmental significance, physiology, and regulation. *Annu. Rev. Microbiol.* 48 311–343. 10.1146/annurev.mi.48.100194.001523 7826009

[B30] Ohnishi S. T. Barr J. K. (1978). A simplified method of quantitating protein using the biuret and phenol reagents. *Anal. Biochem.* 86 193–200. 10.1016/0003-2697(78)90334-2 26278

[B31] Paquete C. M. Turner D. L. Louro R. O. Xavier A. V. Catarino T. (2007). Thermodynamic and kinetic characterisation of individual haems in multicentre cytochromes *c*_3_. *Biochim. Biophys. Acta - Bioenerg.* 1767 1169–1179. 10.1016/j.bbabio.2007.06.005 17692816

[B32] Pettersen E. F. Goddard T. D. Huang C. C. Meng E. C. Couch G. S. Croll T. I. (2021). UCSF ChimeraX: Structure visualization for researchers, educators, and developers. *Protein Sci.* 30 70–82. 10.1002/pro.3943 32881101 PMC7737788

[B33] Pierattelli R. Banci L. Turner D. L. (1996). Indirect determination of magnetic susceptibility tensors in peroxidases: a novel approach to structure elucidation by NMR. *J. Biol. Inorg. Chem.* 1 320–329. 10.1007/s007750050060

[B34] Pokkuluri P. R. Londer Y. Y. Duke N. E. C. Erickson J. Pessanha M. Salgueiro C. A. (2004a). Structure of a novel *c*_7_ -type three-heme cytochrome domain from a multidomain cytochrome *c* polymer. *Protein Sci.* 13 1684–1692. 10.1110/ps.04626204 15133162 PMC2279975

[B35] Pokkuluri P. R. Londer Y. Y. Duke N. E. C. Long W. C. Schiffer M. (2004b). Family of Cytochrome *c*_7_-Type Proteins from *Geobacter sulfurreducens*: structure of One Cytochrome *c*_7_ at 1.45 Å Resolution. *Biochemistry* 43 849–859. 10.1021/bi0301439 14744127

[B36] Pokkuluri P. R. Londer Y. Y. Duke N. E. C. Pessanha M. Yang X. Orshonsky V. (2011). Structure of a novel dodecaheme cytochrome *c* from *Geobacter sulfurreducens* reveals an extended 12nm protein with interacting hemes. *J. Struct. Biol.* 174 223–233. 10.1016/j.jsb.2010.11.022 21130881

[B37] Pokkuluri P. R. Londer Y. Y. Yang X. Duke N. E. C. Erickson J. Orshonsky V. (2010). Structural characterization of a family of cytochromes *c*_7_ involved in Fe(III) respiration by *Geobacter sulfurreducens*. *Biochim. Biophys. Acta Bioenerg.* 1797 222–232. 10.1016/j.bbabio.2009.10.007 19857457

[B38] Popescu D.-M. Newman D. K. Banfiel J. F. (2002). Geomicrobiology: how Molecular-Scale Interactions. *Science* 296 1071–1077. 10.1126/science.1010716 12004119

[B39] Rimboud M. Desmond-Le Quemener E. Erable B. Bouchez T. Bergel A. (2015). Multi-system Nernst-Michaelis-Menten model applied to bioanodes formed from sewage sludge. *Bioresour. Technol.* 195 162–169. 10.1016/j.biortech.2015.05.069 26027903

[B40] Sievers F. Higgins D. G. (2018). Clustal Omega for making accurate alignments of many protein sequences. *Protein Sci.* 27 135–145. 10.1002/pro.3290 28884485 PMC5734385

[B41] Speers A. M. Reguera G. (2012). Electron donors supporting growth and electroactivity of *Geobacter sulfurreducens* anode biofilms. *Appl. Environ. Microbiol.* 78 437–444. 10.1128/AEM.06782-11 22101036 PMC3255729

[B42] Ueki T. (2021). Cytochromes in Extracellular Electron Transfer in *Geobacter*. *Appl. Environ. Microbiol.* 87 1–16. 10.1128/AEM.03109-20 33741623 PMC8117768

[B43] Vasyliv O. M. Bilyy O. I. Ferensovych Y. P. Hnatush S. O. (2013). “Application of acetate, lactate, and fumarate as electron donors in microbial fuel cell,” in *Reliab. Photovolt. Cells, Modul. Components, Syst. VI* 8825, 88250Q, Proc. SPIE 8825. 10.1117/12.2021381

[B44] Wei Y. Thyparambil A. A. Latour R. A. (2014). Protein helical structure determination using CD spectroscopy for solutions with strong background absorbance from 190 to 230 nm. *Biochim. Biophys. Acta Proteins Proteomics* 1844 2331–2337. 10.1016/j.bbapap.2014.10.001 25308773 PMC4395514

[B45] Whitmore L. Wallace B. (2004). A. DICHROWEB, an online server for protein secondary structure analyses from circular dichroism spectroscopic data. *Nucleic Acids Res.* 32 W668–W673. 10.1093/nar/gkh371 15215473 PMC441509

[B46] Yoho R. A. Popat S. C. Torres C. I. (2014). Dynamic potential-dependent electron transport pathway shifts in anode biofilms of *Geobacter sulfurreducens*. *Chem. Sus. Chem.* 7 3413–3419. 10.1002/cssc.201402589 25351488

[B47] Zacharoff L. Chan C. H. Bond D. R. (2016). Reduction of low potential electron acceptors requires the CbcL inner membrane cytochrome of *Geobacter sulfurreducens*. *Bioelectrochemistry* 107 7–13. 10.1016/j.bioelechem.2015.08.003 26407054

