# Supplementary material for: Electron Flow From the Inner Membrane Towards the Cell Exterior in Geobacter sulfurreducens: Biochemical Characterization of Cytochrome CbcL
Source: Front Microbiol. 2022 May 10;13:898015. doi: 10.3389/fmicb.2022.898015 (PMC9129911; doi:10.3389/fmicb.2022.898015)
Supplement: Supplementary file 1 [file Data_Sheet_1.pdf]

Supplementary Information

Electron flow from the inner membrane towards the cell exterior in *Geobacter sulfurreducens*: biochemical characterization of cytochrome CbcL

Jorge M. A. Antunes[1,2], Marta A. Silva[1,2], Carlos A. Salgueiro[1,2,*], Leonor Morgado[1,2,*]

[1]Associate Laboratory i4HB - Institute for Health and Bioeconomy, NOVA School of Science and Technology, NOVA University Lisbon, 2829-516 Caparica, Portugal

[2]UCIBIO – Applied Molecular Biosciences Unit, Chemistry Department, NOVA School of Science and Technology, NOVA University Lisbon, 2829-516 Caparica, Portugal

[*]Corresponding authors: csalgueiro@fct.unl.pt, mlmorgado@fct.unl.pt

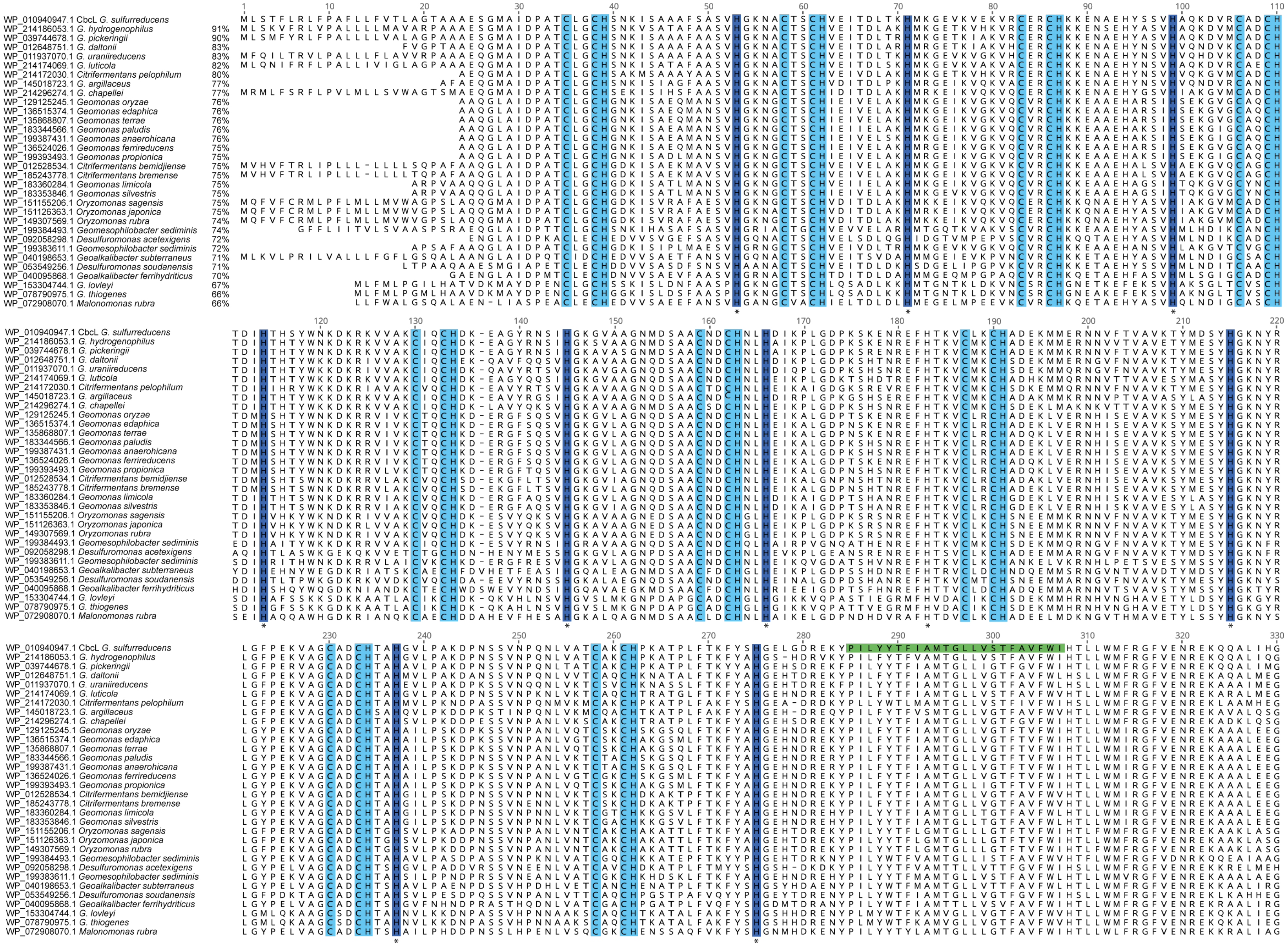

Supplementary Figure 1 – Analysis of CbcL amino acid sequence. Alignment of the full-length CbcL sequence and homologous proteins with pairwise identity above 65% as retrieved by BLAST (Altschul et al., 1997) and aligned with Clustal Omega (Sievers and Higgins, 2018). NCBI access number and species are identified for each sequence. The heme binding motifs CXXCH are colored in light blue, the conserved histidine residues in the periplasmic domain are marked with a * and those which are axial ligands are colored in dark blue. The residues in the transmembrane helices are colored in green (as predicted by TMHMM - 2.0; Möller et al., 2001).

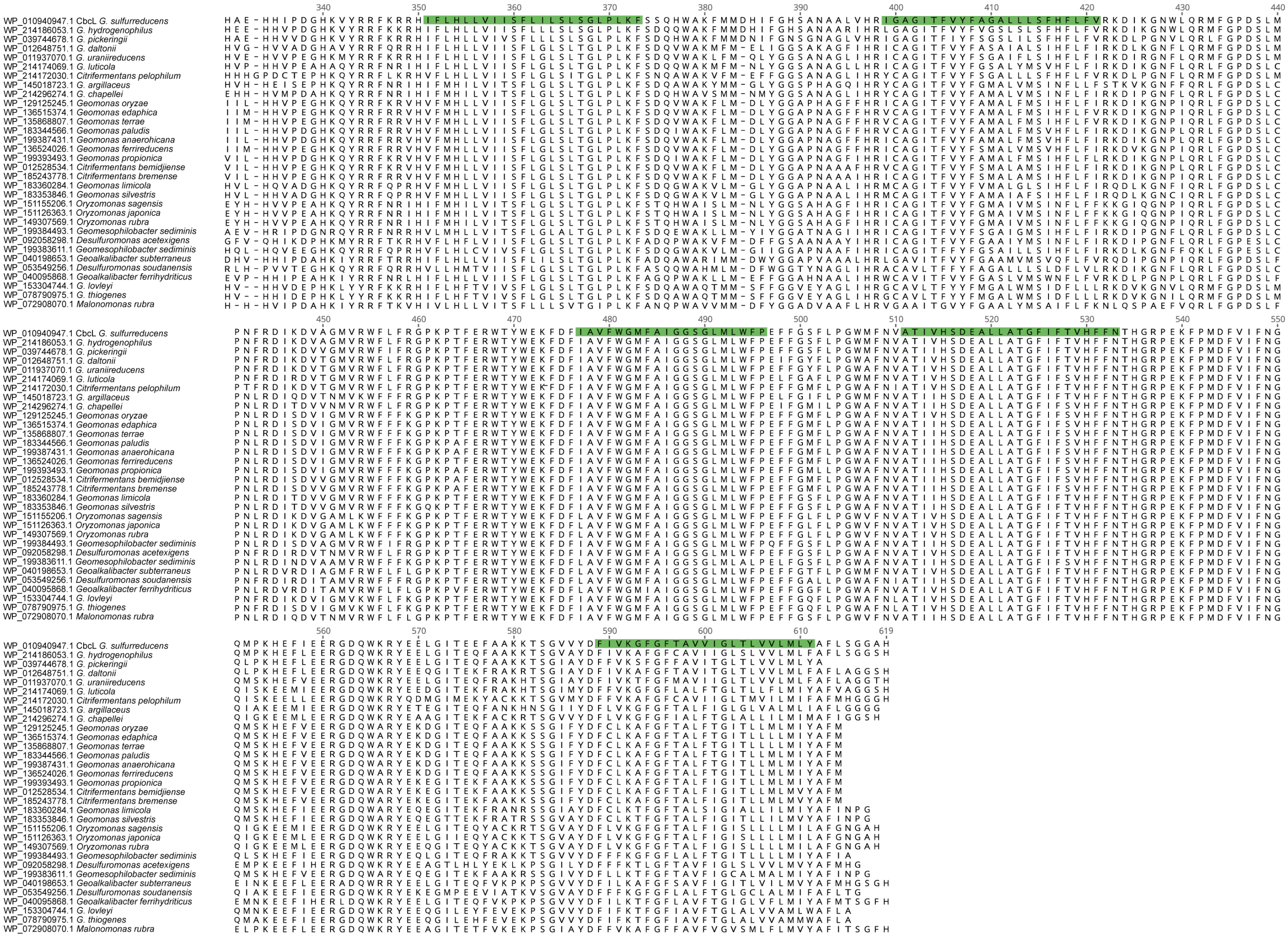

Supplementary Figure 1 – CbcL amino acid sequence analysis (continued).

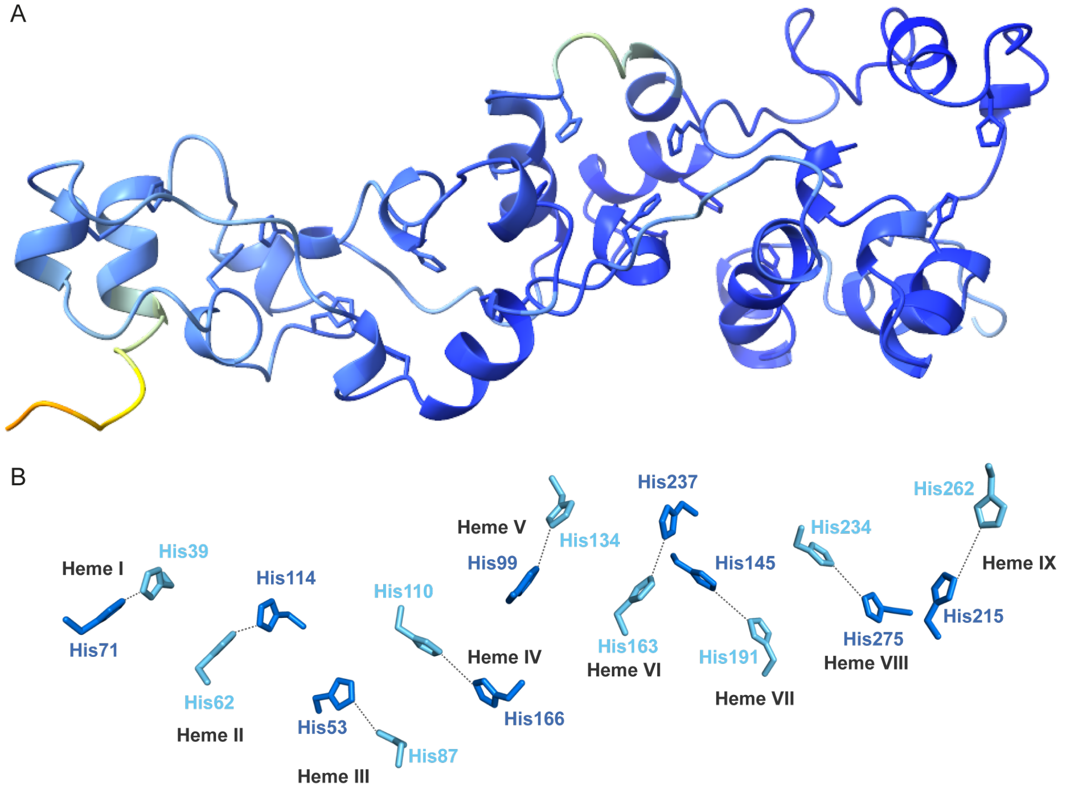

Supplementary Figure 2 – AlphaFold prediction of CbcL periplasmic domain structure (Jumper et al., 2021). (A) Structural model in cartoon representation colored by prediction confidence (B-factor). (B) Heme axial ligands. Histidine residues from the heme binding motifs CXXCH are colored in light blue (proximal ligands) and distal ligands in dark blue.

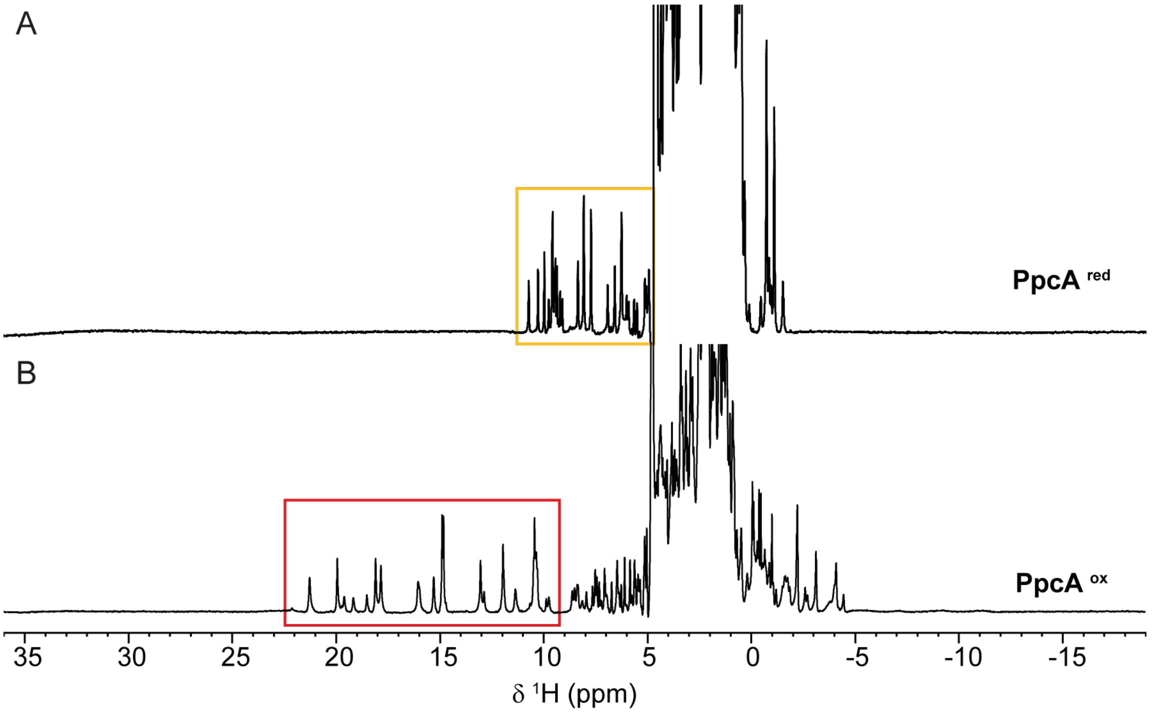

Supplementary Figure 3 – 1D ^{1}H NMR spectrum of cytochrome PpcA in the reduced (A) and oxidized (B) forms. Spectra were acquired at 25 °C with 100 μM of PpcA in 10 mM sodium phosphate pH 8. The yellow and red rectangles highlight the PpcA fingerprints in the reduced and oxidized states, respectively.

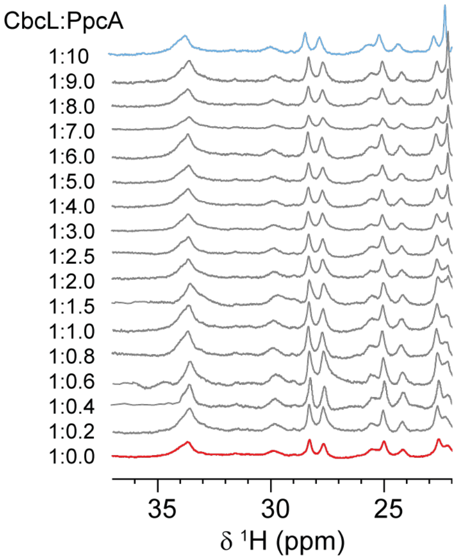

Supplementary Figure 4 – NMR chemical shift perturbation experiments of CbcL in the presence of PpcA. 1D ^{1}H NMR spectra of CbcL acquired with increasing amounts of PpcA (ratio CbcL:PpcA indicated on the left side of each spectrum). Spectra were acquired at 25 °C with 100 μM of CbcL in 10 mM sodium phosphate pH 8.

References

Altschul, S. F., Madden, T. L., Schäffer, A. A., Zhang, J., Zhang, Z., Miller, W., et al. (1997). Gapped BLAST and PSI-BLAST: A new generation of protein database search programs. *Nucleic Acids Res.* 25, 3389–3402. doi:10.1093/nar/25.17.3389.

Jumper, J., Evans, R., Pritzel, A., Green, T., Figurnov, M., Ronneberger, O., et al. (2021). Highly accurate protein structure prediction with AlphaFold. *Nature* 596, 583–589. doi:10.1038/s41586-021-03819-2.

Möller, S., Croning, M. D. R., and Apweiler, R. (2001). Evaluation of methods for the prediction of membrane spanning regions. *Bioinformatics* 17, 646–653. doi:10.1093/BIOINFORMATICS/17.7.646.

Sievers, F., and Higgins, D. G. (2018). Clustal Omega for making accurate alignments of many protein sequences. *Protein Sci.* 27, 135–145. doi:10.1002/pro.3290.
