# Supplementary material for: Electron Flow From the Inner Membrane Towards the Cell Exterior in Geobacter sulfurreducens: Biochemical Characterization of Cytochrome CbcL
Source: Front Microbiol. 2022 May 10;13:898015. doi: 10.3389/fmicb.2022.898015 (PMC9129911; doi:10.3389/fmicb.2022.898015)
Supplement: Supplementary file 2 [file Presentation_1.zip › CbcL_Figure5.pdf]

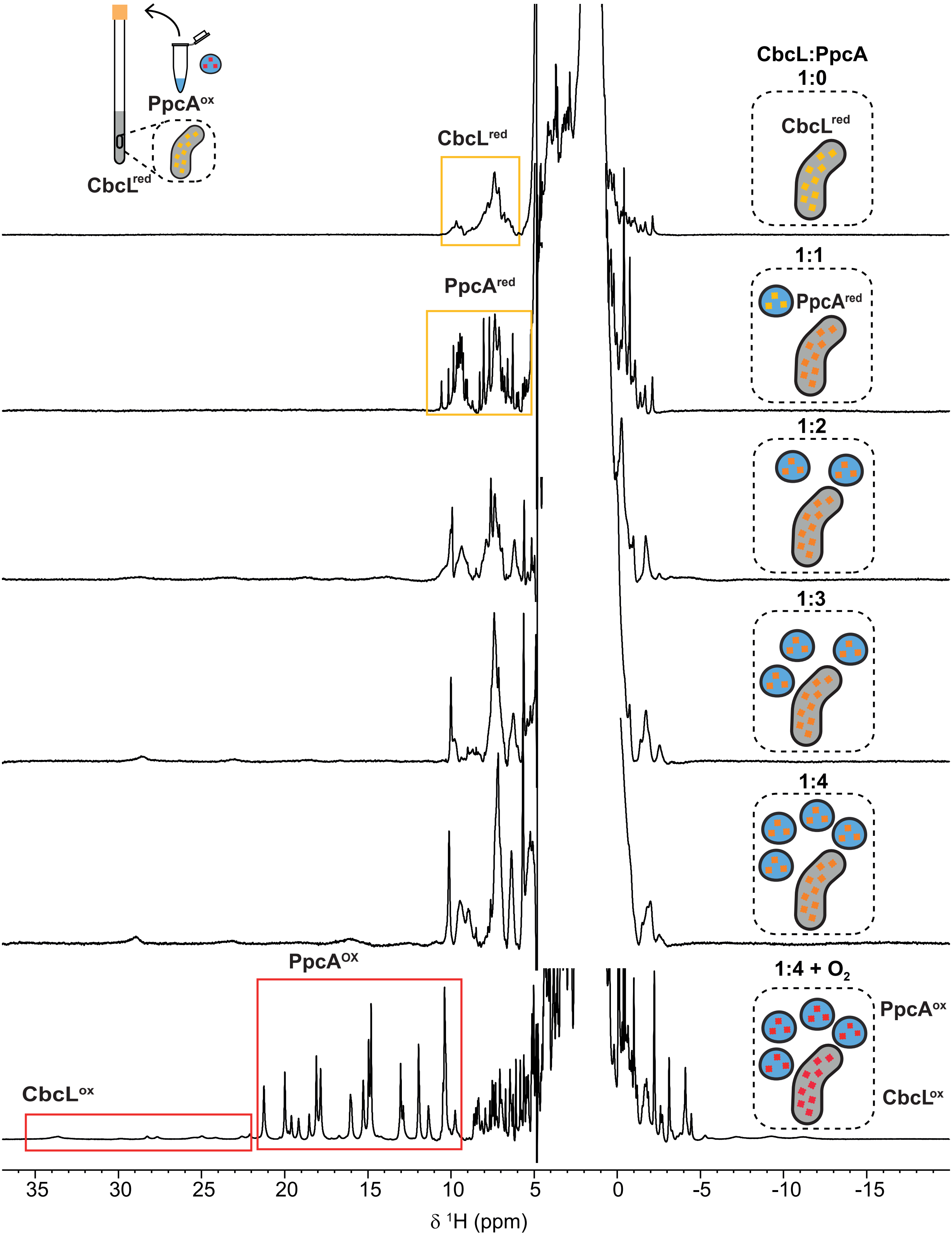

PpcA$^{ox}$
CbcL$^{red}$
CbcL:PpcA
1:0
CbcL$^{red}$
CbcL$^{red}$
1:1
PpcA$^{red}$
PpcA$^{red}$
1:2
1:3
1:4
PpcA$^{ox}$
1:4 + $O_2$
PpcA$^{ox}$
CbcL$^{ox}$
CbcL$^{ox}$
35 30 25 20 15 10 5 0 -5 -10 -15
δ $^1$H (ppm)
