# Supplementary figures and images for: Electron Flow From the Inner Membrane Towards the Cell Exterior in Geobacter sulfurreducens: Biochemical Characterization of Cytochrome CbcL
Source: Front Microbiol. 2022 May 10;13:898015. doi: 10.3389/fmicb.2022.898015 (PMC9129911; doi:10.3389/fmicb.2022.898015)

A

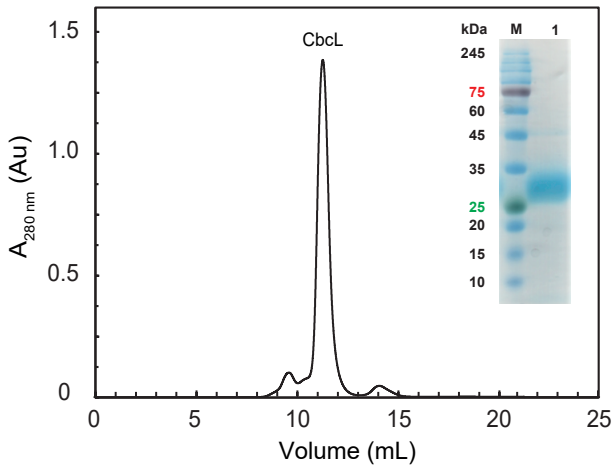

B

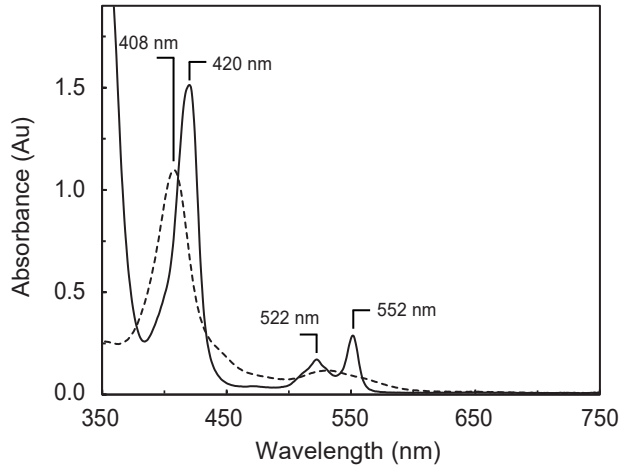

Supplement: Supplementary file 2 [file Presentation_1.zip › CbcL_Figure1.pdf]

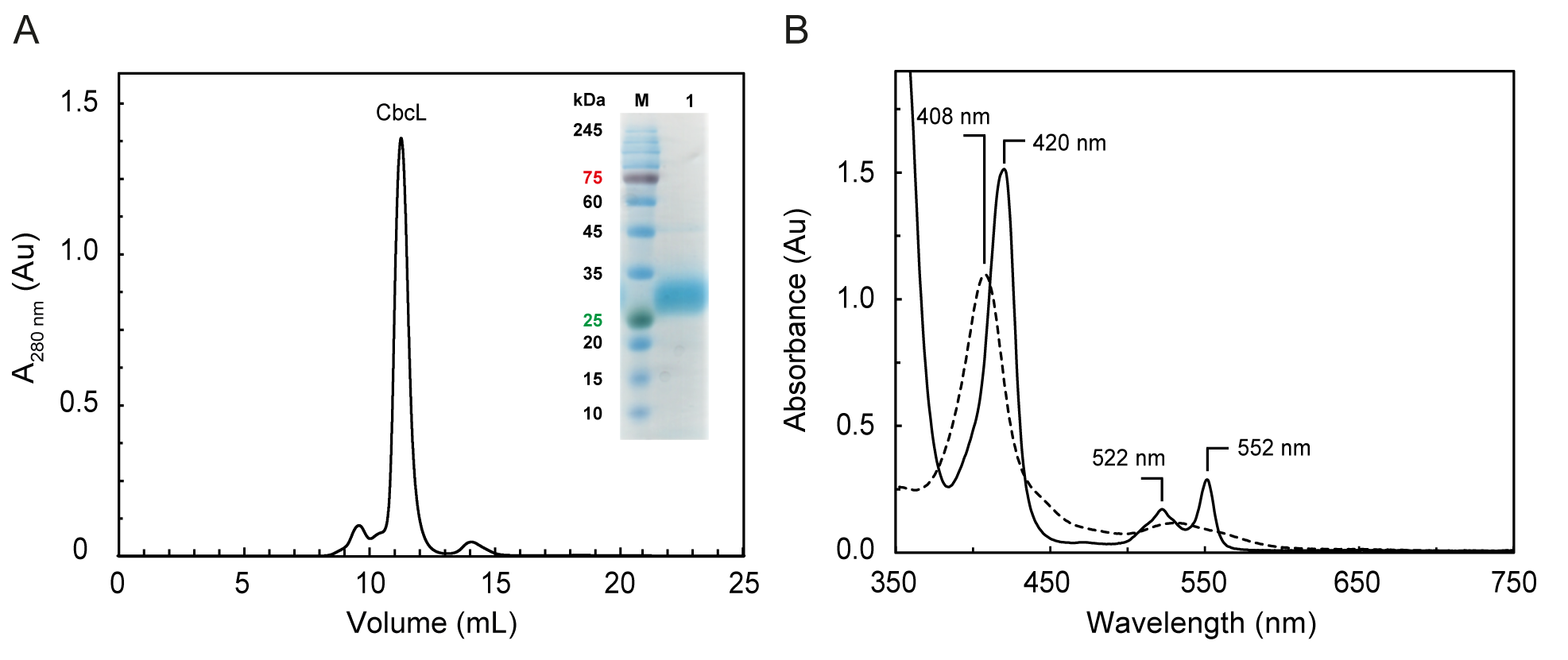

Supplement: Supplementary file 2 [file Presentation_1.zip › CbcL_Figure1.png]

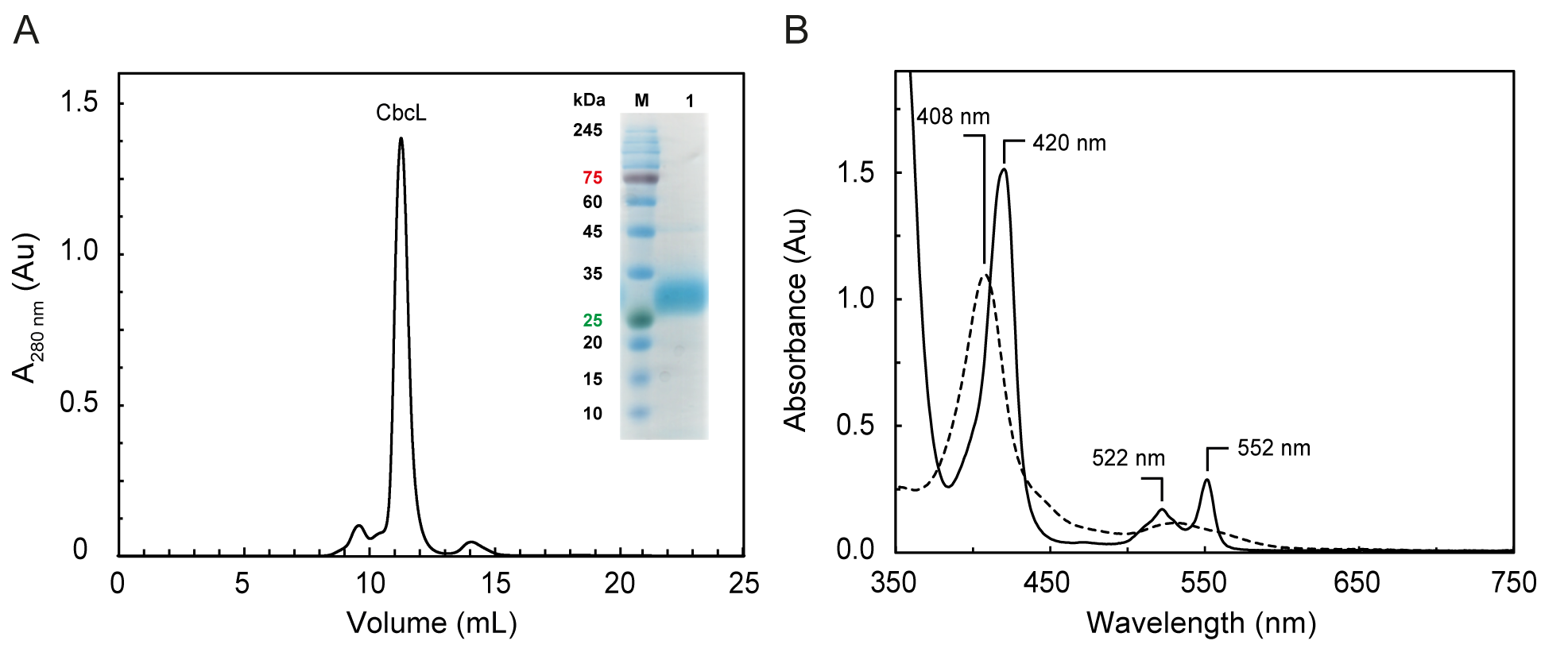

Supplement: Supplementary file 2 [file Presentation_1.zip › CbcL_Figure1.tif]

A

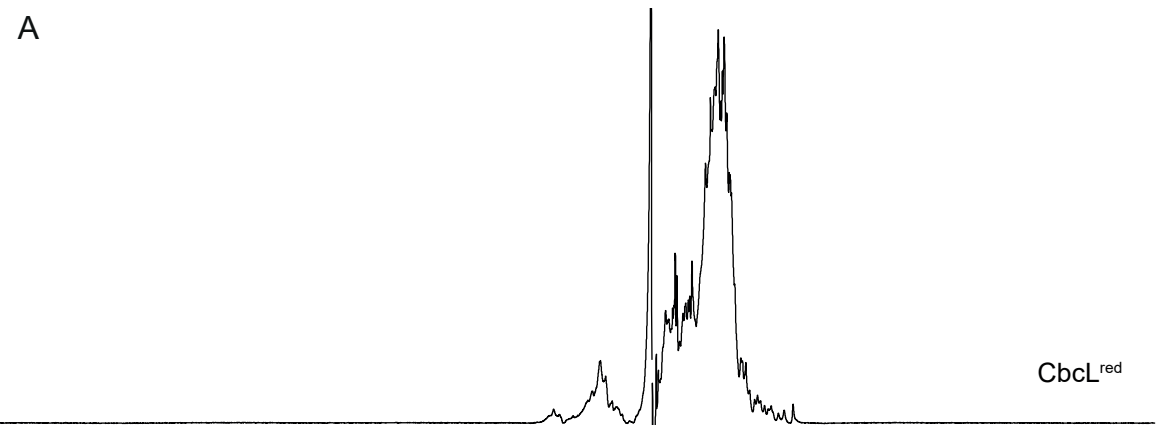CbcL<sup>red</sup>

B

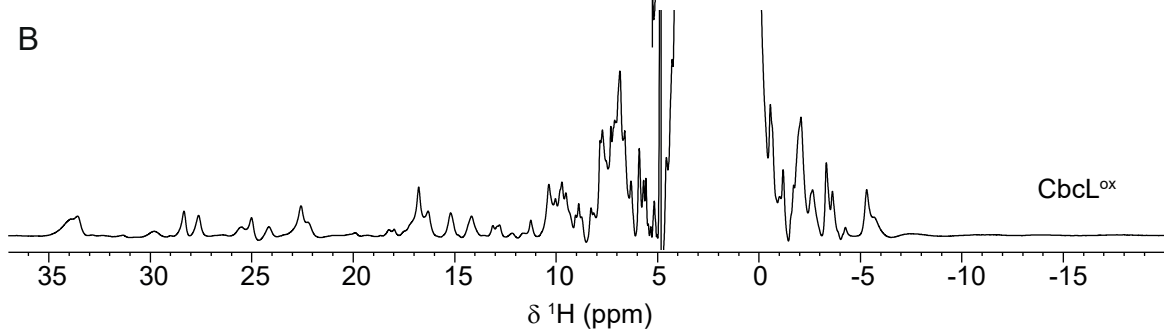CbcL<sup>ox</sup>

Supplement: Supplementary file 2 [file Presentation_1.zip › CbcL_Figure2.pdf]

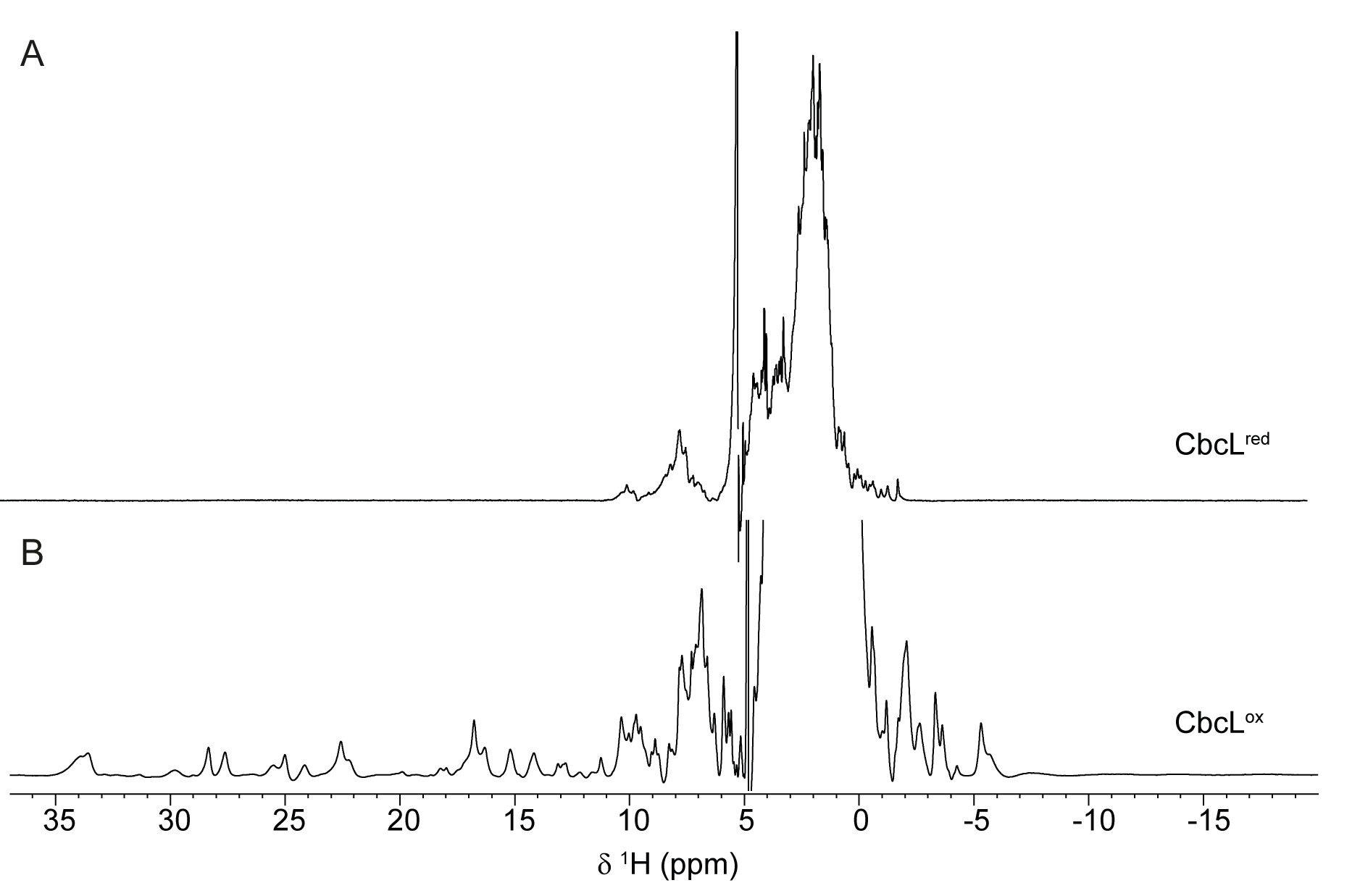

Supplement: Supplementary file 2 [file Presentation_1.zip › CbcL_Figure2.png]

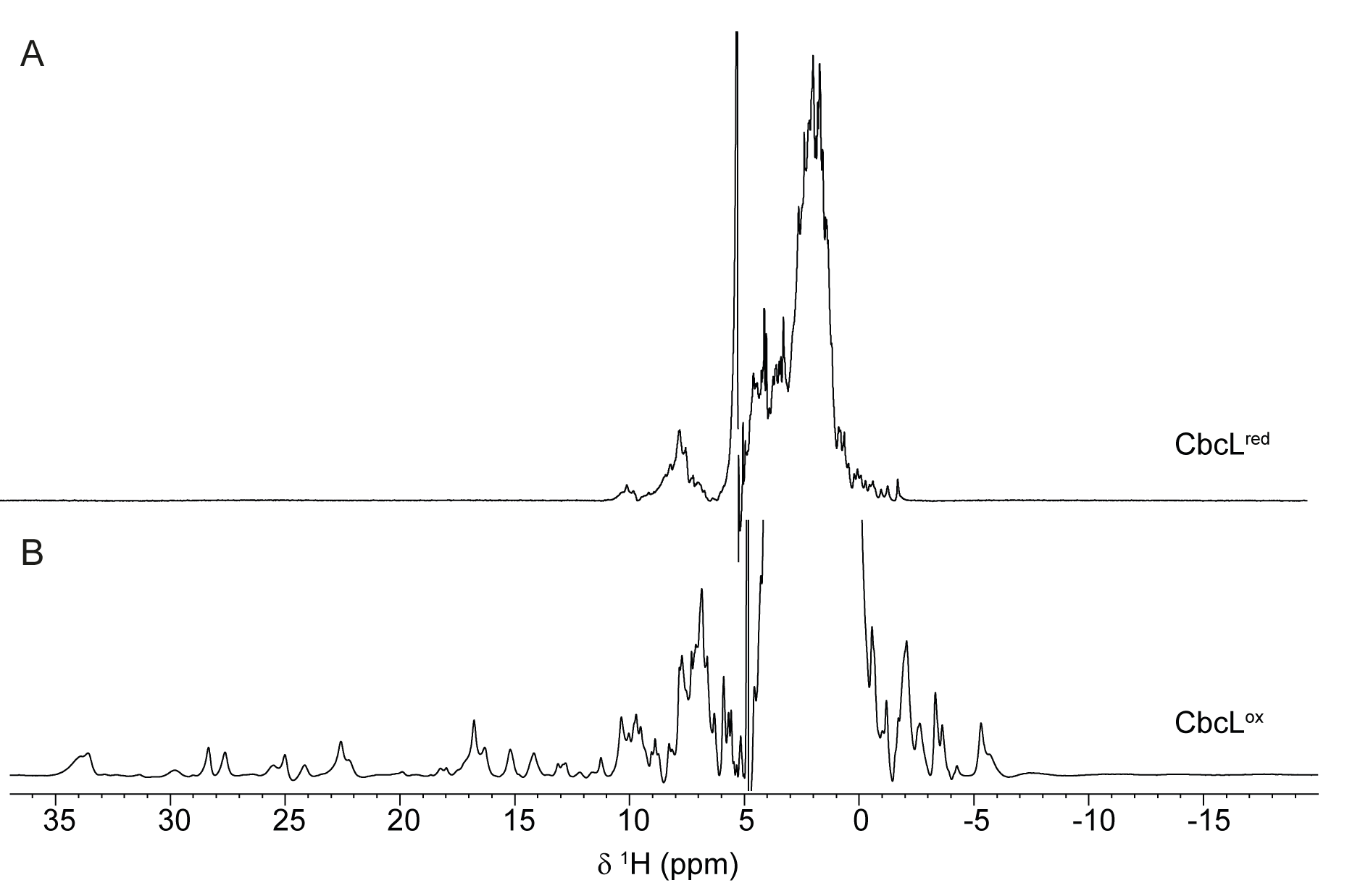

Supplement: Supplementary file 2 [file Presentation_1.zip › CbcL_Figure2.tif]

**A**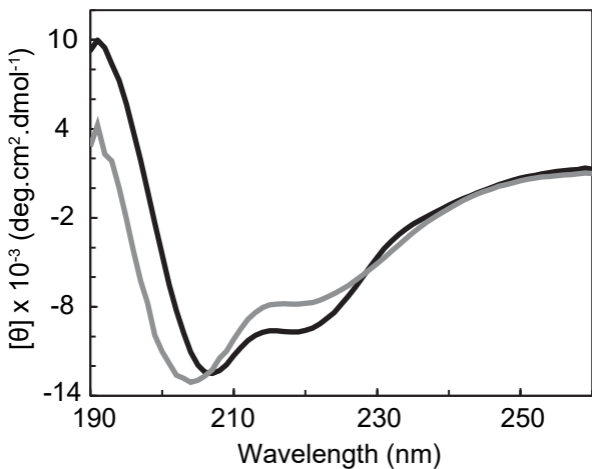**B**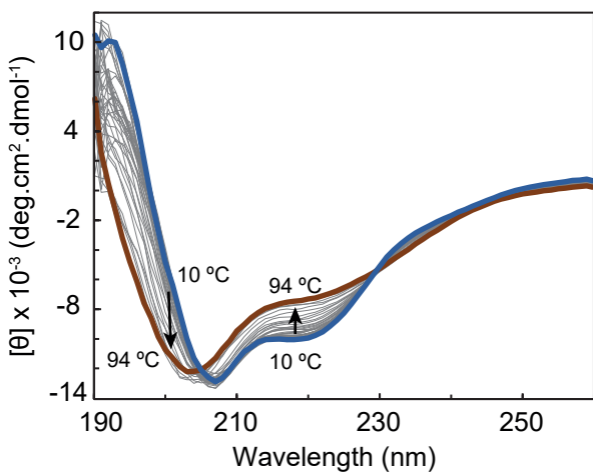**C**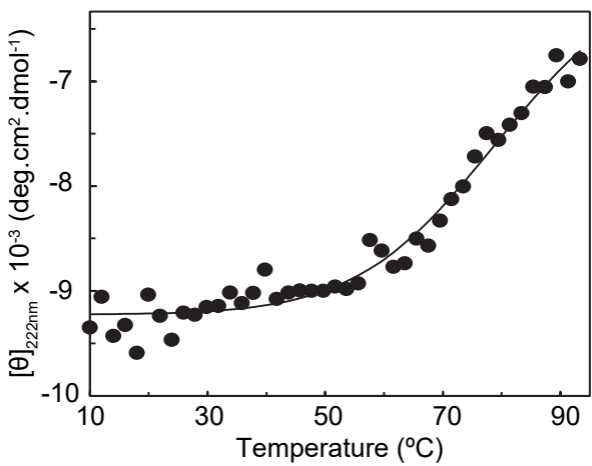

Supplement: Supplementary file 2 [file Presentation_1.zip › CbcL_Figure3.pdf]

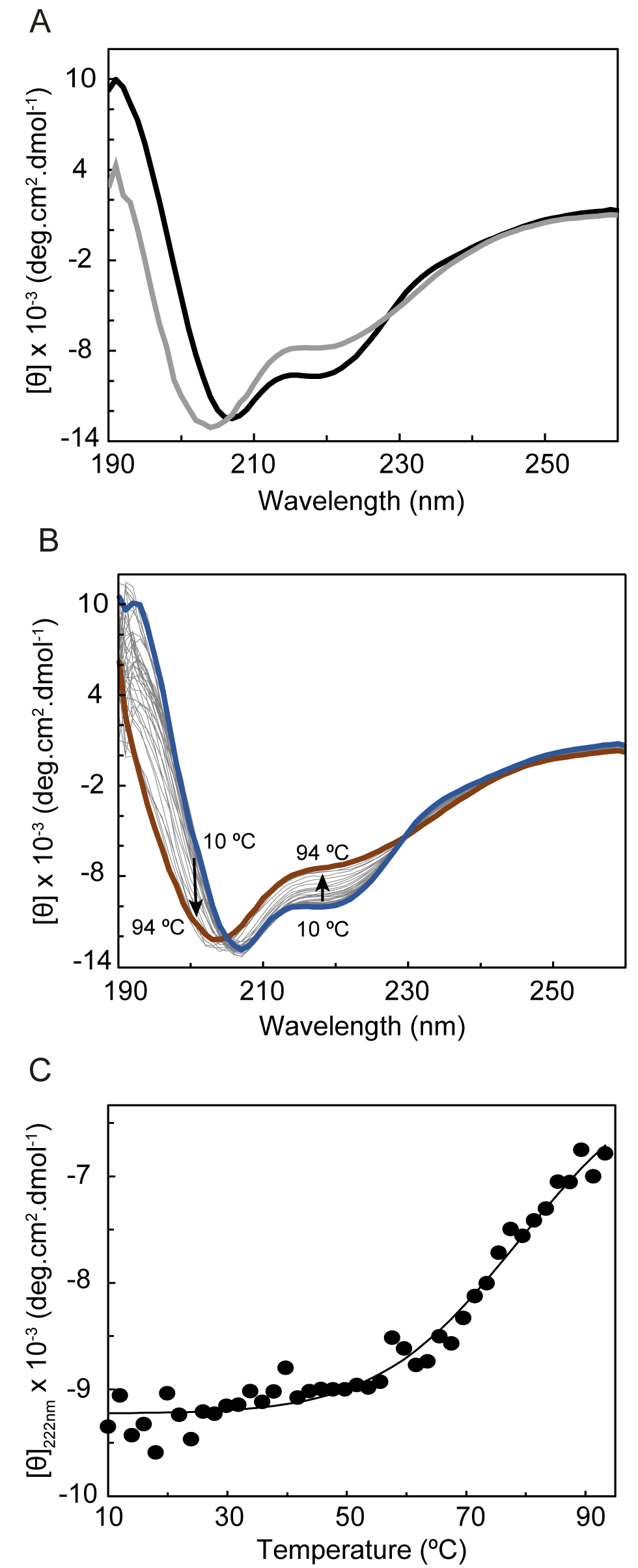

Supplement: Supplementary file 2 [file Presentation_1.zip › CbcL_Figure3.png]

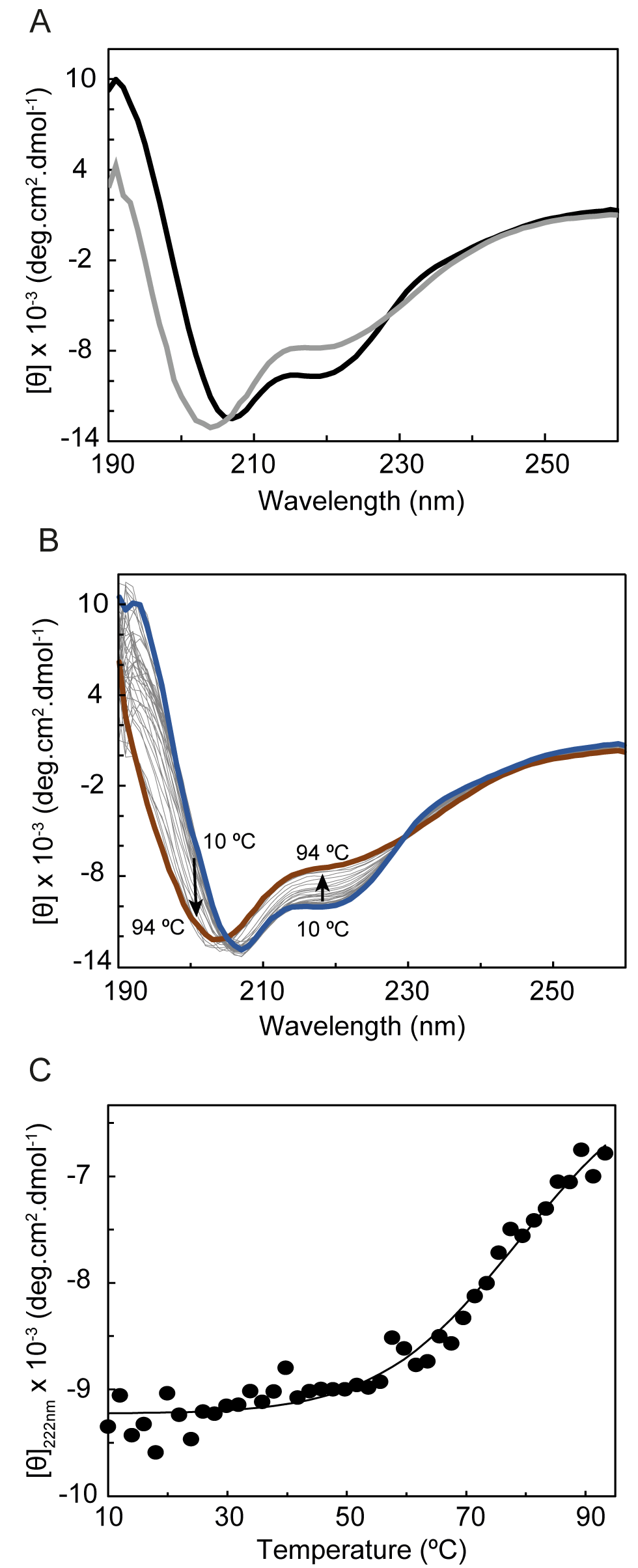

Supplement: Supplementary file 2 [file Presentation_1.zip › CbcL_Figure3.tif]

A

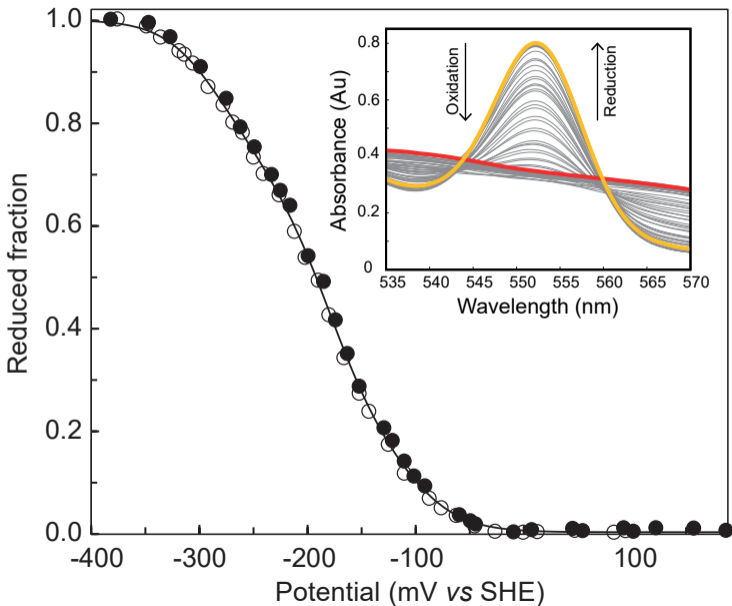

B

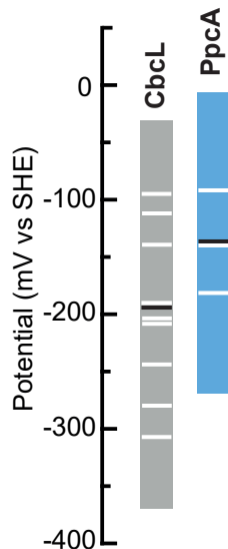

C

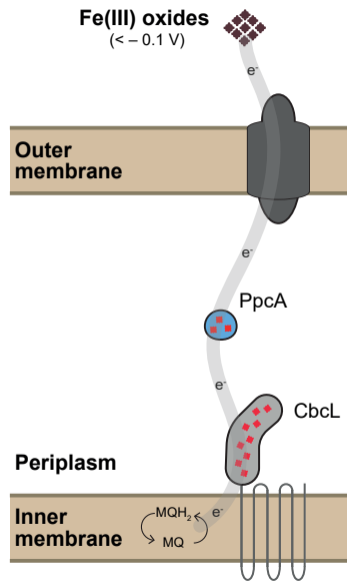

Supplement: Supplementary file 2 [file Presentation_1.zip › CbcL_Figure4.pdf]

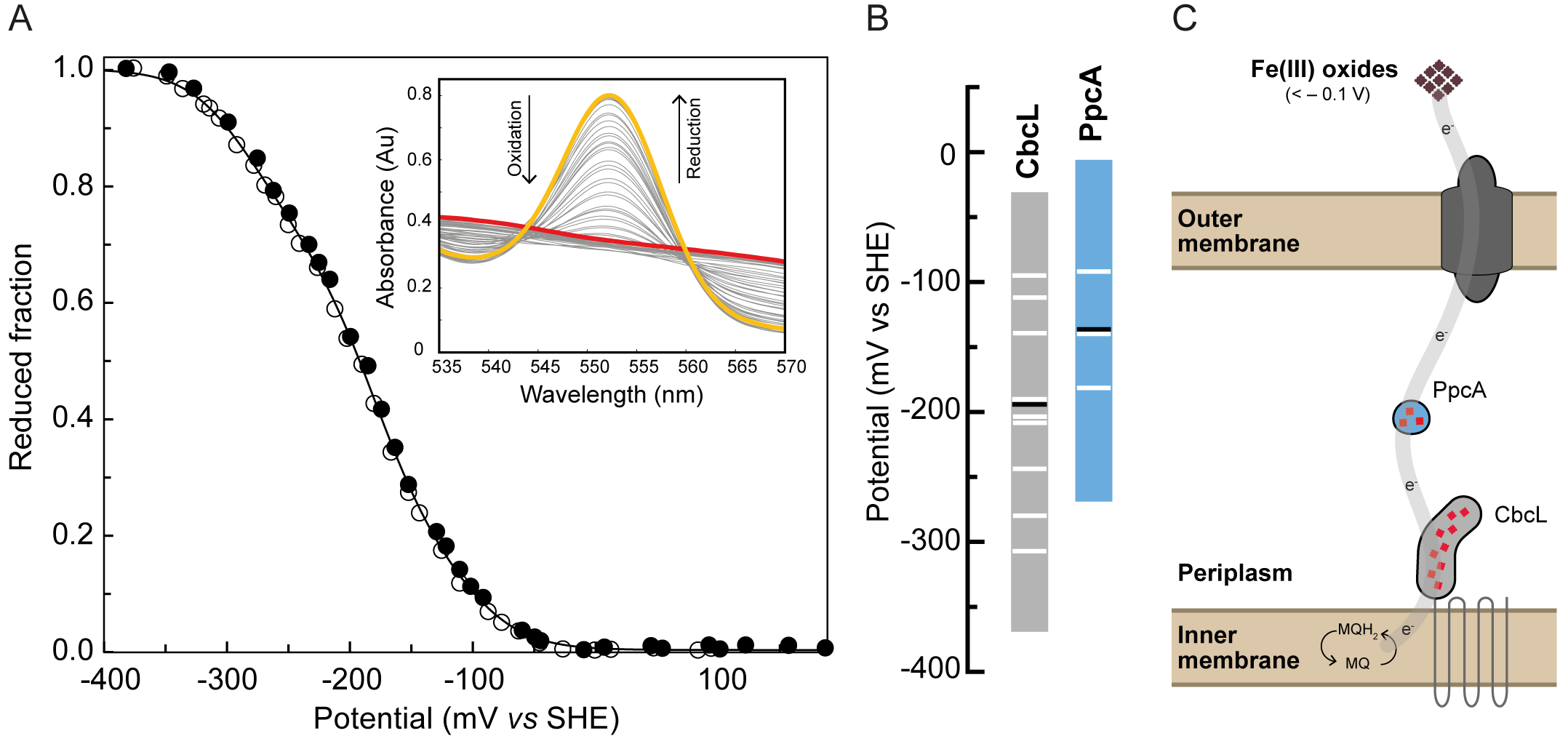

Supplement: Supplementary file 2 [file Presentation_1.zip › CbcL_Figure4.png]

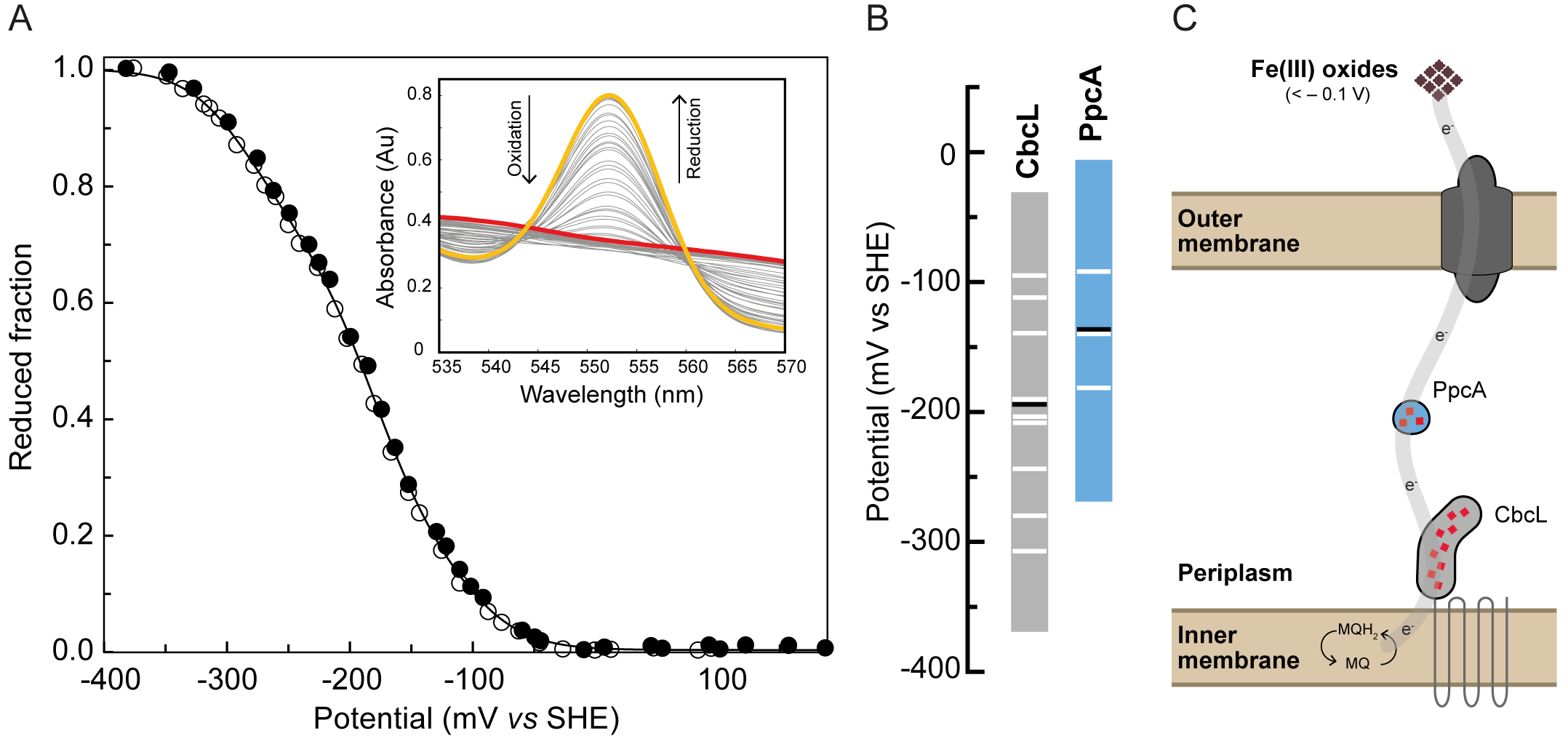

Supplement: Supplementary file 2 [file Presentation_1.zip › CbcL_Figure4.tif]

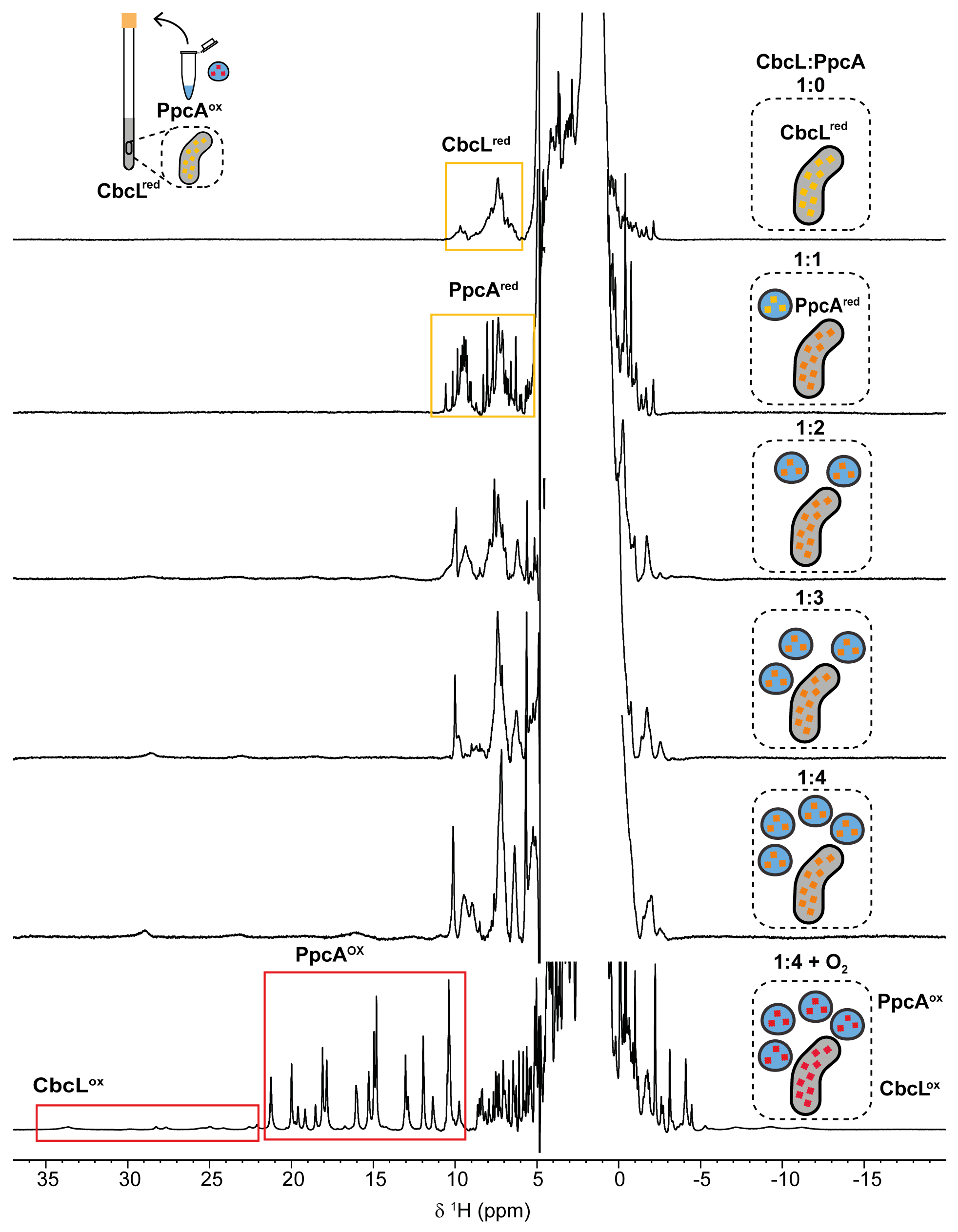

Supplement: Supplementary file 2 [file Presentation_1.zip › CbcL_Figure5.png]

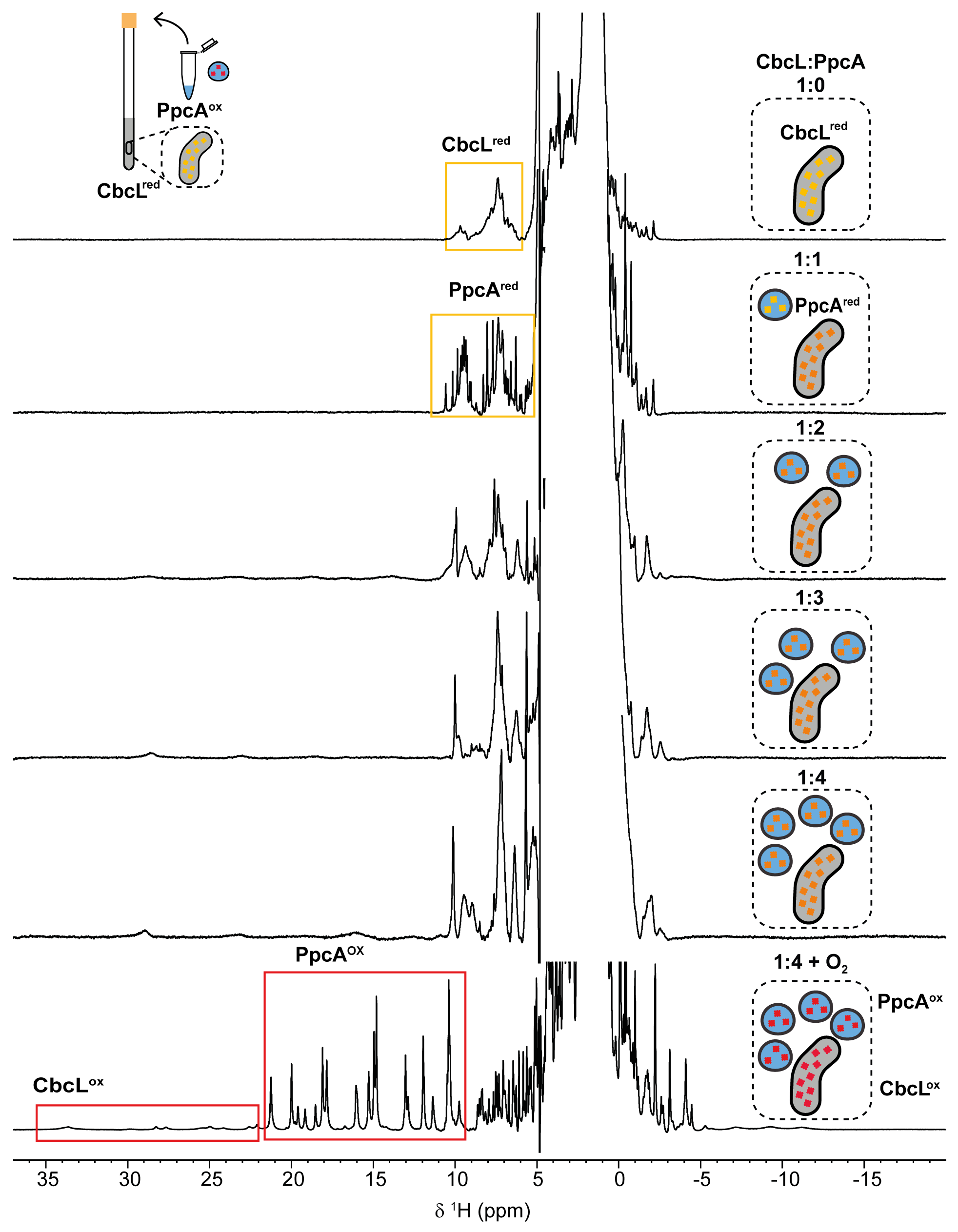

Supplement: Supplementary file 2 [file Presentation_1.zip › CbcL_Figure5.tif]

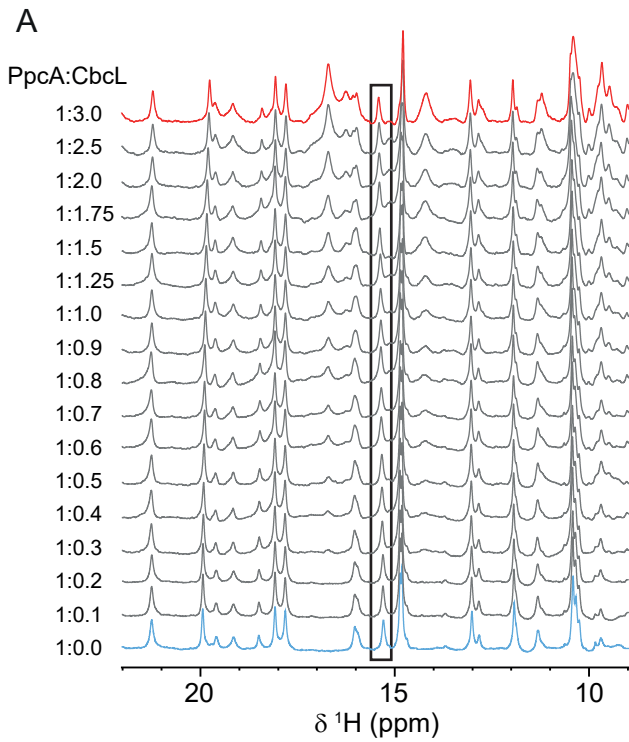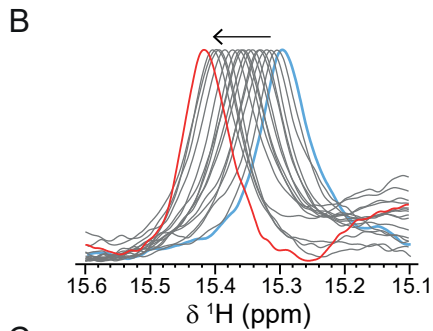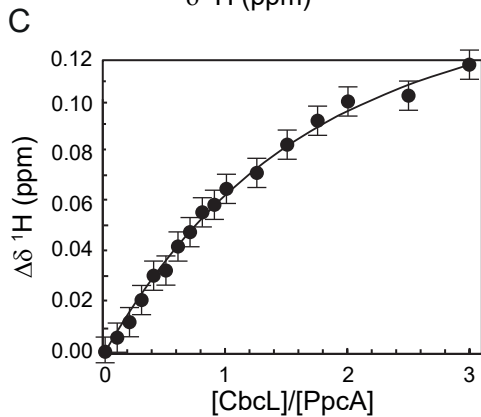

Supplement: Supplementary file 2 [file Presentation_1.zip › CbcL_Figure6.pdf]

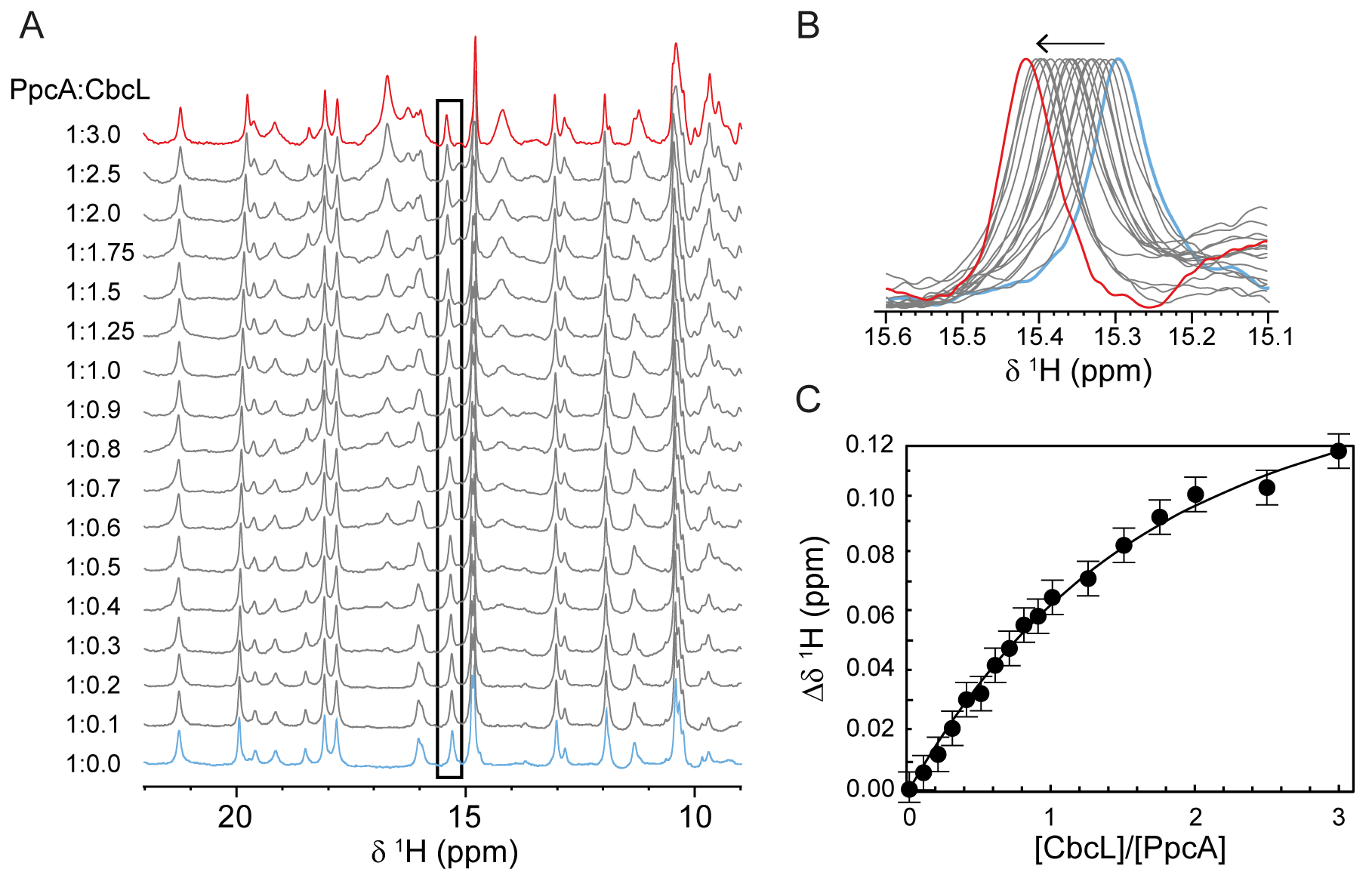

Supplement: Supplementary file 2 [file Presentation_1.zip › CbcL_Figure6.png]

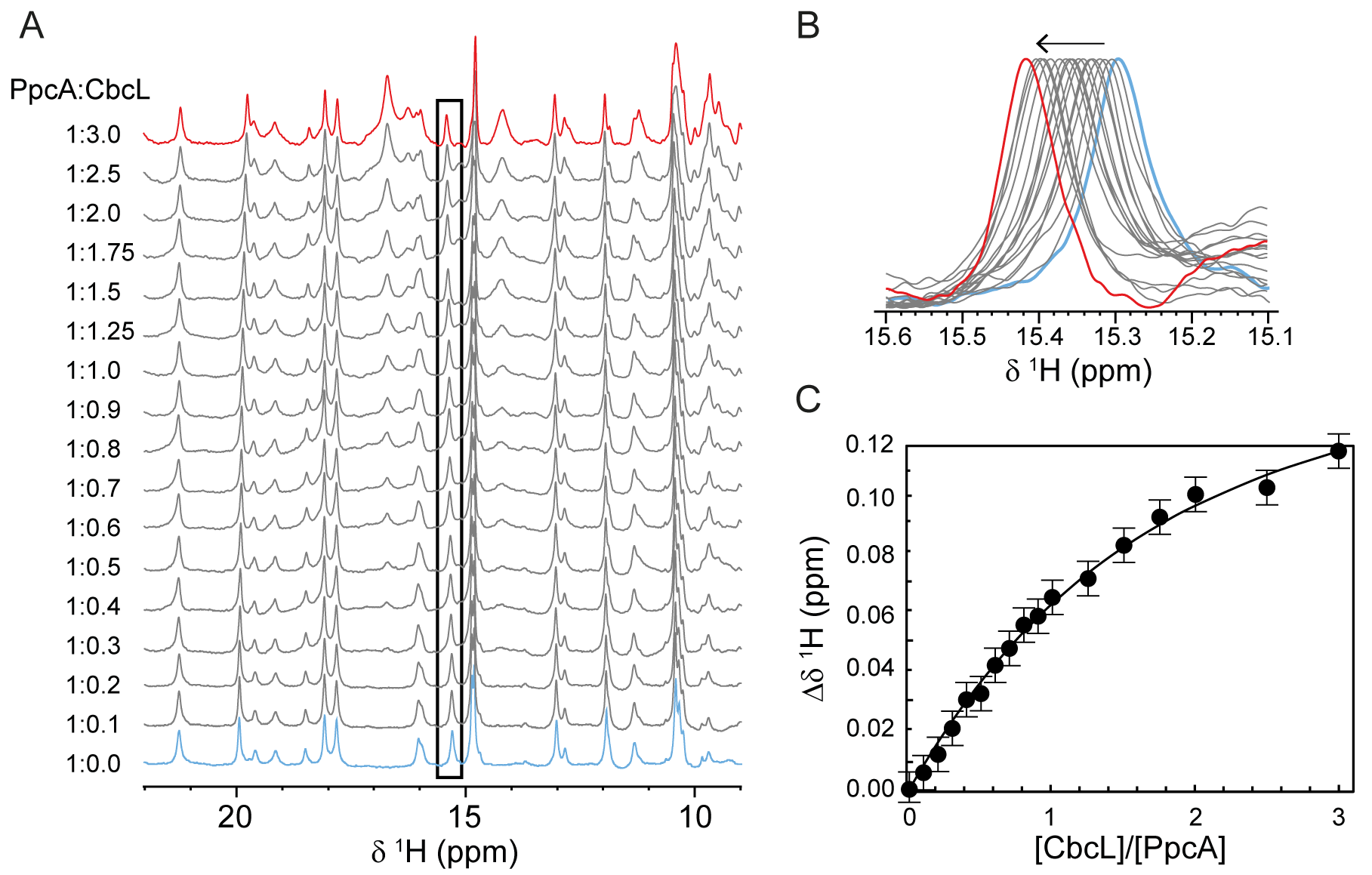

Supplement: Supplementary file 2 [file Presentation_1.zip › CbcL_Figure6.tif]
